# Laser Synthesis and Microfabrication of Micro/Nanostructured Materials Toward Energy Conversion and Storage

**DOI:** 10.1007/s40820-020-00577-0

**Published:** 2021-01-04

**Authors:** Lili Zhao, Zhen Liu, Duo Chen, Fan Liu, Zhiyuan Yang, Xiao Li, Haohai Yu, Hong Liu, Weijia Zhou

**Affiliations:** 1 Collaborative Innovation Center of Technology and Equipment for Biological Diagnosis and Therapy in Universities of Shandong, Institute for Advanced Interdisciplinary Research (iAIR), University of Jinan, Jinan, 250022 People’s Republic of China; 2 State Key Laboratory of Crystal Materials, Shandong University, Jinan, 250100 People’s Republic of China; 3 School of Information Science and Engineering, Shandong University, 72 Binhai Road, Qingdao, 266237 People’s Republic of China

**Keywords:** Laser synthesis, Laser microfabrication, Micro/nanostructured materials, Energy conversion and storage

## Abstract

Nanomaterials are known to exhibit a number of interesting physical and chemical properties for various applications, including energy conversion and storage, nanoscale electronics, sensors and actuators, photonics devices and even for biomedical purposes. In the past decade, laser as a synthetic technique and laser as a microfabrication technique facilitated nanomaterial preparation and nanostructure construction, including the laser processing-induced carbon and non-carbon nanomaterials, hierarchical structure construction, patterning, heteroatom doping, sputtering etching, and so on. The laser-induced nanomaterials and nanostructures have extended broad applications in electronic devices, such as light–thermal conversion, batteries, supercapacitors, sensor devices, actuators and electrocatalytic electrodes. Here, the recent developments in the laser synthesis of carbon-based and non-carbon-based nanomaterials are comprehensively summarized. An extensive overview on laser-enabled electronic devices for various applications is depicted. With the rapid progress made in the research on nanomaterial preparation through laser synthesis and laser microfabrication technologies, laser synthesis and microfabrication toward energy conversion and storage will undergo fast development.

## Highlights

- The current understanding and advances on laser synthesis of nanomaterials are summarized.
- The laser microfabrication-enabled energy conversion and storage devices are reviewed.
- The limitations and solutions for current laser processing of nanomaterials and other more potential development directions for laser processing are proposed.

## Graphical abstract

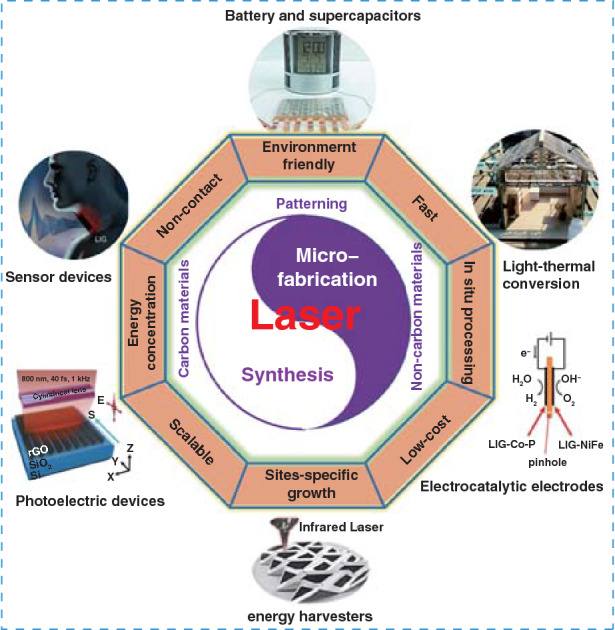

## Introduction

Nanomaterials have presented a number of interesting physical and chemical properties for various applications, including energy storage and conversion [1], nanoscale electronics [2], sensors and actuators [3], photonics devices [4], and even for biomedical purposes [5]. The researches on nanomaterial synthesis have a long history, and a large number of different synthesis approaches have been performed up until now, including the wet chemical method in solution conducting environment and the thermal treatment process in gas conducting environment. However, uniform and large-scale production of nanomaterials remains a challenge, and the nanomaterials synthesized through these conventional approaches are non-site specific in general. Thus, new technologies for large-scale production and position controllability are necessary.

The laser as a synthetic technique and laser as a microfabrication technique provide the alternative choice, which has the advantages of fast, scalable, environment friendly, cost-effective and permitting in situ processing [6, 7]. Compared with the traditional synthesis and microfabrication techniques, laser-assisted processing techniques enable direct synthesis of nanomaterials in both gas environment and liquid environment with environment friendliness and less energy loss; especially, the suitability for processing of thermally sensitive substrates has more advantages. On the other hand, recent advances in laser microfabrication incorporate mask unemployment for more advanced patterns of nanomaterials. Specially, as the synthetic technique, the traditional wet chemical methods are capable of producing nanomaterials with unique morphologies, but toxic or environmentally unfriendly reagents were frequently used. On the contrary, laser synthesis methods in solution conducting environment commonly used the target as precursor, avoiding the use of toxic reagents, and nanomaterials with smaller particle sizes can be obtained through regulation of the laser power, laser wavelength, laser focal length, laser pulse width and laser frequency [8]. Thermal treatment or annealing process is also frequently used methods for nanomaterial synthesis, which is conducted in the furnace at a high temperature by depending on the material formation thermodynamics. But this procedure undergoes some issues of time consuming, high thermal power and energy loss with sample dimensions considerably smaller than the heated volume. In addition, such methods are not suitable for thermally sensitive substrates such as ITO glass or polymers, where microstructural changes and thermal-expansion mismatch will occur under high temperatures. Alternatively, the laser synthesis technique with a focused irradiation beam enables a site-specific growth of nanomaterials based on the local photo-thermo-chemical reaction, which possesses the general advantages of spatially confined reaction, non-contact, fast processing speeds by direct writing and 3D compatibility [9]. The advantage of spatially confined reaction for laser synthesis technology means that the laser-induced photothermal effect or photochemical effect through synthesis of the purpose materials at a localized position to construct the patterned nanostructures. This can be called the spatially confined reaction by laser, which is strongly dependent on the laser process parameters, especially the pulse width. In addition, additional selectivity and unique properties of nanomaterials for some special applications can be conveyed by regulating the applied laser parameters [10]. As the microfabrication technique, employing masks for the definition of patterns on substrates and lithographic techniques are essential for the conventional fabrication method, which are awkward and expensive for constructing various devices for applications. The laser microfabrication technique obviates the need for masks and lithography, thereby enhancing the production yield of devices and promoting flexibility in the design of device geometry [11]. In conclusion, the laser provides not only an effective alternative to the conventional synthesis and microfabrication processes, but also innovative selective synthesis and microfabrication strategies for efficient usage of nanomaterials with minimized environmental requirements and damage on the substrate.

**Scheme 1:**
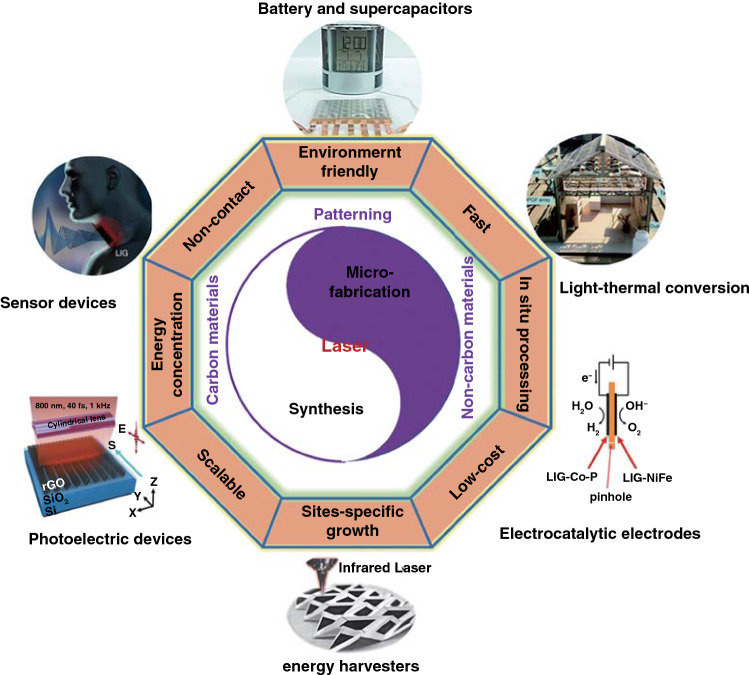
Laser as a synthetic technique and a microfabrication technique and their applications in various functional devices

Herein, this review focuses on the nanostructures and nanomaterials using laser as synthesis and microfabrication technique, which are applied in energy conversion and storage. As well known, the absorption of laser by the precursor materials and then resulting in various effects, such as melting, plasma formation and vaporization, are the basis of laser processing of materials. As the consequence, the characteristics of the laser (laser intensity, wavelength and pulse width) and the photo-thermophysical properties of the precursors determine the extent of these effects. In this review, we initially discuss laser processing as the synthetic technique for nanomaterial synthesis, including carbon nanomaterials and non-carbon nanomaterials. Subsequently, we provide a comprehensive overview on the laser as a microfabrication technique applied in light–thermal conversion, batteries and supercapacitors, sensors, or actuators and electrocatalytic electrodes (Scheme 1). Finally, recent progress and advances in laser synthesis and microfabrication processing of nanomaterials for applications in various devices are discussed.

## Laser as the Synthetic Technique

Recent technological advances in the development of diverse lasers have opened new avenues in material synthesis, no matter in solid form or in solution environment. The laser technique has long been utilized for material synthesis, which is realized by the photothermal reaction, photochemical reaction or photo-thermal-chemical reaction derived from an irradiated laser by generating confined electromagnetic field at a desired position with high controllability.

### Carbon Nanomaterials

Among all of the synthesis methods, annealing or thermal treatment of the precursors is usually necessary to obtain the carbon nanomaterials [12, 13]. Conventionally, the thermal treatment of polymer precursors is conducted in a furnace or an oven at a high temperature, which suffer from a quite slow cooling rate and high energy loss due to the smaller sample dimensions than the heated volume. In addition, the morphology and structure of the precursors were difficult to inherit or create new nanostructures. As an alternative to the conventional annealing, laser processing offers a potential solution to the above issues and enables construction of different nanostructures from the precursors to carbon materials, on account of the localized thermal effect without any interference with the surrounding materials during laser processing [14].

Various kinds of carbon nanomaterials can be derived through laser processing technologies, including graphene-related material, diamond-like carbon, glassy carbon, and heteroatom-doped carbon. Specially, graphene-based structures have become a rising star owing to its unique physical and chemical properties [15, 16], which can be obtained from different precursors by laser processing, such as graphene oxide, polymers, CH_4_, SiC and so on. In this section, the recent progress in the laser processing of different carbon materials from various precursors is reviewed.

#### Laser Synthesis of Graphene

##### Laser Synthesis of Graphene from Graphene Oxide

**Fig. 1:**
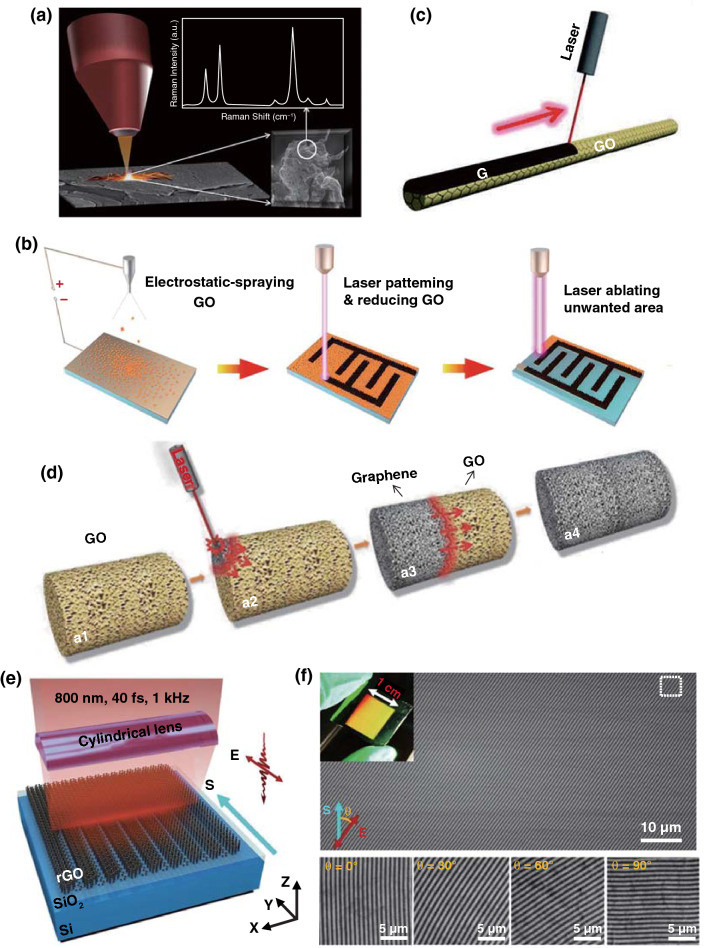
**a** Reduction of GO by laser irradiation utilized **a** Nd-YAG laser (pulse width in the range of ms) with an excitation wavelength of 1064 nm [21]. **b** Schematic illustration of ultrathin laser-processed graphene-based micro-planar supercapacitors. After the different powers of laser treatment, the reduction and ablation of GO film were completed [22]. **c** Representation of positioned laser reduction on one side of a GO fiber. The black region corresponds to the laser-induced G region along the brown GO fiber [23]. **d** Schematic illustration of the preparation process of graphene bulks and functional counterparts induced by a laser shot within milliseconds [24]. **e** Schematic of grating processing of a GO film using cylindrical focusing of femtosecond laser pulses. **f** Photograph (insert) and SEM image of the large-area (10 × 12 mm^2^) rGO. *θ* represents the angle between S and E [28]

Graphene oxide (GO) has been widely utilized as the precursors for synthesizing the reduced graphene oxide (rGO) to remove the oxygen-containing groups by thermal reduction at a high temperature [17], chemical reduction by means of reductants [18], and photoreduction with light irradiation [19]. Among these, the laser synthesis of rGO has been demonstrated to be an efficient and facile approach for reduction of graphene oxide into graphene and provides an opportunity for localized reduction and controllable patterning [20]. Yannopoulos et al. compared the thermal reduction, chemical reduction, and laser-assisted reduction approaches of GO to rGO. It turned out that high-quality graphene-like structures with efficient defect healing and ultralow sheet resistance were produced by the laser-induced reduction of GO (Fig. 1a) [21], while the thermal reduction and chemical reduction methods cannot provide a high conductivity for the as-prepared rGO. This was because that the high temperature induced by laser (> 2500 °C) was able not only to remove oxygen-containing species but also in favor of eliminating the structural defects in the formed rGO, which would result in a high conductivity for the rGO by laser-assisted reduction method. Wong et al. [22] reduced the electro-sprayed GO thin film into rGO and ablated the unwanted areas by using a 355 nm nanosecond laser. After tuning the output laser power, the patterned electrode arrays were completed. (Fig. 1b). In addition, Qu et al. fabricated an asymmetric Graphene/Graphene oxide fiber through the laser-assisted partial reduction of GO fibers with graphene in the scanned region and GO in the unexposed region (Fig. 1c). The obtained asymmetric G/GO fiber was an ideal material as a moisture-sensitive fiber actuator [23]. Thus, the laser synthesis of rGO possessed the advantage of controllable reduction of partial GO to rGO. This group further reported a spontaneous reduction of GO aerogel strategy by laser triggering (Fig. 1d). Pure graphene, doped graphene, and multifunctional graphene composited metals or metal oxides with macroscopic bulk structure have been prepared by this fast solvent- and reagent-free preparation method [24]. Only within 37.5 ms, a 5 cm^3^ GO aerogel was completely reduced to graphene bulk. Unlike the dense structure of GO fiber in Ref. [23] or dense GO film in Ref. [22], where only the exposed region of GO was reduced to the rGO, the fewer stacked graphene sheets within the pore-rich structure of GO aerogel and the available air stored in the pores played the crucial roles for the sustainable reduction of GO.

As for the solid GO film typically suffers from the restacking as a result of the strong van der Waals interactions, which caused a low specific capacitance. The LightScribe laser of DVDs can simultaneously cause reduction and exfoliation of GO and then produce an open network of laser-scribed graphene (LSG). For instance, Kaner [25] used a Light Scribe DVD optical drive to perform on graphite oxide films, the graphene sheets were achieved and restacking was avoided. It was realized by a drop-cast GO thin film on a flexible substrate and then irradiating with an infrared laser inside a Light Scribe DVD optical drive. During the laser LightScribe process, the GO was reduced into the well-exfoliated LSG sheets, which can be indicated by the change in film color and the SEM images. Kaner et al. [26] also used a LightScribe DVD burner to fabricate graphene micro-supercapacitors over large areas by direct laser writing on graphite oxide films. Thanks to the precision of a laser, the desired graphene circuits were produced under the driving of the computer-designed pattern onto the GO film. Large scale and patterning were realized within a short time. Luo et al. [27] also used the scribing process for the reduction and patterning of graphene oxide film to realize the LSG sensors fabrication. This will be discussed in detail in Sects. 3.2 and 3.3.

As well known, laser reduction of GO conforms to the mechanisms of photochemical reduction under a short-wavelength laser (e.g., UV laser) and photothermal reduction under a relative long-wavelength laser (e.g., Vis and NIR laser). Recently, Guo et al. [28] proposed that by utilizing the transverse electric (TE) surface plasmons triggered by the gradient reduction of the GO film from its surface to the interior, a diverse laser–rGO interaction occurred through producing an inhomogeneous slab with the maximum dielectric permittivity (DP) at the surface and a smaller DP at deeper thicknesses that allows excitation of transverse electric (TE) mode surface plasmons (TE-SPs). This TE-SPs eventually resulted in interference intensity fringes and spatially periodic interactions (Fig. 1e). It was noted that the laser-processed grating structure strictly parallel to the TE polarization state of the incident light. This implies that complex topography preparation is feasible by changing only the polarization (Fig. 1f).

##### Laser Synthesis of Graphene from Polymer

 Besides the GO, polymers were another frequently used carbon source for laser synthesis of graphene, especially for the polyimide (PI). Different from the 2D sheet-like graphene derived from GO by laser, porous graphene with a larger specific surface area can be obtained by laser scribing of polymers. During the conversion process from polymers, the *sp*^3^-carbon atom arrangement of polymers converted to *sp*^2^-carbon atom arrangement under the photothermal effect induced by pulsed laser irradiation. Professor Tour’s group devoted a considerable amount of effort to laser-induced graphene (LIG) conversion from polyimide or the other polymers [29–31]. In 2014 [32], they reported a one-step laser scribing on commercial PI in air to form 3D-graphene layers (CO_2_ laser with a laser power of 3.6 W). The effect of laser power on the formation of LIG was investigated to obtain that the conversion from PI to LIG occurred at the threshold power of 2.4 W. The thermal power dominated the quality of films when the laser power was < 4.2 W, and a higher degree of graphitization occurred with the increase of laser power. When the laser power increased to above 4.2 W, oxidation started to play an increasingly deleterious role in the film quality. In addition, the mechanism for laser scribing of polymers was also thoroughly investigated in this article. It was speculated that photothermal effects owing to the long wavelength (~ 10.6 μm) and relatively long pulse width (~ 14 μs) of the CO_2_ laser caused lattice vibrations, which led to extremely high localized temperatures (˃2500 °C). C–O, C=O and N–C bonds were broken under such high temperatures and atoms recombination occurred to release the corresponding gases, which could be confirmed by the dramatically decreased oxygen and nitrogen contents in the polymer. The author also found out that the mechanism of laser graphitization in polymers was strongly correlated to the structural features of the repeat units, such as aromatic and imide repeat units. Through testing 15 different polymers by laser-induced graphitization process, it demonstrated that only two polymers, PI and poly(etherimide), both of which contain aromatic and imide repeat units, can form LIG, while the other step-growth polymers and the chain-growth polymers cannot afford LIG. However, in 2018 [33], Their further research reported that multiple lasing method allows that any material that can be converted into amorphous carbon can be further treated by a CO_2_ laser beam to obtain graphene, such as Kapton, Kevlar, polysulfones, poly(etherimide), polyphenylene sulfide, phenolic resin, and cross-linked polystyrene. Finally, it can be concluded that the wavelength of the laser irradiation as well as the number of exposures are important to the formation of LIG. Then by means of this laser graphitization from PI technique, Professor Tour’s group successively employed the laser-induced graphene for flexible and embeddable gas sensors [34], and preparing porous B-doped graphene with promising electrochemical performance by introducing H_3_BO_3_ into PI [35]. Recently, Zhang et al. [36] used the laser writing of Kevlar textile in air to prepare a Janus graphene/Kevlar textile. With the motorized and computer-controlled laser writing, LIG could be simply and rapidly written into various geometries from Kevlar textile. Similar to the conversion of PI, the C=O and N−C bonds in Kevlar being broken and the reorganization of remaining carbon atoms into graphene can be ascribed to photothermal effect induced by the laser irradiation.

**Fig. 2:**
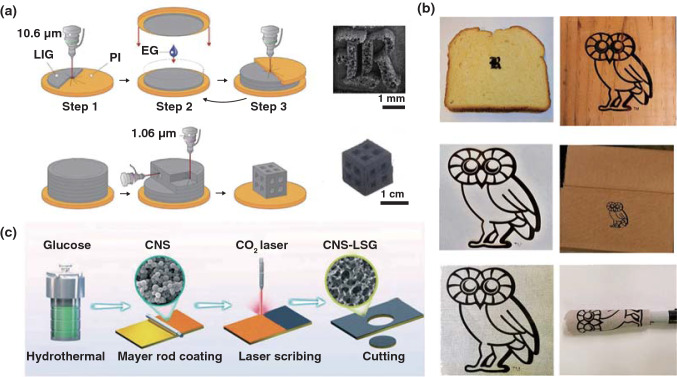
**a** Manufacturing and processing of laser-induced 3D GFs [37]. **b** LIG induced from bread, fire-retardant treated pine wood, cotton paper, cardboard box, gray muslin cloth and muslin cloth wrapped around a marker [33]. **c** Schematic diagram of the laser scribing fabrication of CNS-LSG electrode [42]

Tour’s group [37] also developed a 3D LIG foam by combining the printing process on basis of laminated object manufacturing and the laser treatment process, which break through the limitation of only 2D products on the PI substrate being able to be synthesized by traditional laser-induced graphene. They denoted this process as modified, automated 3D-printing process based on laminated object manufacturing (LOM). During the preparation process, ethylene glycol served as an adhesive through capillary forces between the layers, and stacked PI on top of one another was irradiated by a fiber laser, as shown in Fig. 2a. Except for laser-induced graphene from PI, Professor Tour’s group also demonstrated a three-dimensional (3D) printing graphene foam (GF) by using a mixture of Ni and sucrose as precursor [38], which was also realized by in situ manually adding multiple layers of a mixture of Ni and sucrose to overcome the limitation of microsized scales due to the precursors contained in inkjet-printable or UV-curable inks for the traditional 3D printing technique. Graphene was formed under the template and catalyst effects of Ni and sucrose as carbon source. Besides, various naturally occurring substrates (Fig. 2b) such as paper, cloth, coconut shells, potato skins, cork [33] and polytetrafluoroethylene (Teflon, or PTFE) [39] have been tried to prepare LIG by this group. Some other groups also have done further researches on LIG based on the work of Tour’s group [40]. Luo et al. [41] prepared laser-induced graphene paper (LIGP) with different shapes and structures. Similarly, they confirmed that the polymer precursor with considerable spaces was beneficial to a deep absorption of thermal energy resulted from laser and thus was conducive to the formation of LIGP. Alshareef et al. [42] used glucose-derived amorphous carbon nanospheres (CNS) as carbon source, and a highly turbostratic graphitic carbon electrode was fabricated by laser scribing, which was composed of a 3D framework structure dominated by meso- and macro-pores (Fig. 2c).

In conclusion, the polymer precursor for laser processing is a more appropriate approach to prepare porous and 3D graphene, which is realized by photothermal effects induced by laser. Thus, the CO_2_ laser with a long wavelength (~ 10.6 μm) is preferable choice when using polymers as precursors. CO_2_ lasers are unique compared to ordinary industrial lasers in that they usually have a wavelength of ~ 10.6 μm when output in the form of long-wave infrared. Many organic materials, including paper, wood, plastics, rubber, textiles, and leather, are highly absorbent for such long wavelengths. The mechanism for laser scribing of polymers was speculated that photothermal effects owing to the long wavelength of the CO_2_ laser caused lattice vibrations, which led to the breaking of C–O, C=O, and N–C bonds and atoms recombination to form LIG. As reported [43], polyimide was ablated with a 308 nm XeCl excimer laser, and the carbon material was characterized after 200−800 pulses, but no graphene-based 2D Raman peaks were detected. By contrast, a CO_2_ laser could yield LIG from a wide variety of substrates for only 3−5 passes processing. Hence, the wavelength of the laser irradiation is important to the formation of LIG. Furthermore, laser-induced graphene from polymer precursors presents a novel strategy for simultaneous graphene formation and patterning.

As mentioned above, the localized high temperature induced by CO_2_ laser can be used to selectively convert some carbon precursors into graphene materials. In fact, the laser-induced graphitization comprises various photothermal reactions, photochemical reactions, or thermal accumulation effect from high repetition rate laser. In terms of thermal accumulation effect, because the time interval between sequential laser pulses is significantly shorter than the time required for heat to diffuse out of the focal volume, hence the energy from successive laser pulses accumulates in and around the focal volume over time. Wang et al. [44] utilized the high-repetition picosecond laser to processing the bakelite plate, which provides great potential to stimulate both research and industrial interest in the development of bakelite-derived carbon materials. It demonstrated that the heat accumulation effect was critical for laser-induced transient heating of bakelite materials, and the subsequent instantaneous high temperature field localized in the microscale domain to generate the hierarchically macropores and mesopores. Young-Jin Kim et al. [45] reported the direct laser writing of graphene oxide patterns using femtosecond laser pulses with different repetition rates. The results showed that the thermal accumulation effects dominantly affected the reduction degree and the linewidth of treated GO. Consequently, it can be concluded that, photochemical reduction under a short-wavelength laser and photothermal reduction under a relative long-wavelength laser are commonly used for laser-induced graphene. But the graphene produced by these two mechanisms is generally treated with nanosecond lasers, compared with ultra-short ps and fs lasers, nanosecond laser caused the low level of achievable precision for patterns. Fortunately, thanks to the recent development of fs and ps laser sources operating at high repetition rates of hundreds kHz up to a few MHz, the development of thermal accumulation effects is expected to be an alternative in LIG, which can achieve the high-conductivity graphene under the premise of ensuring the precision of patterns.

##### Laser Synthesis of Graphene from CH_4_ and SiC

**Fig. 3:**
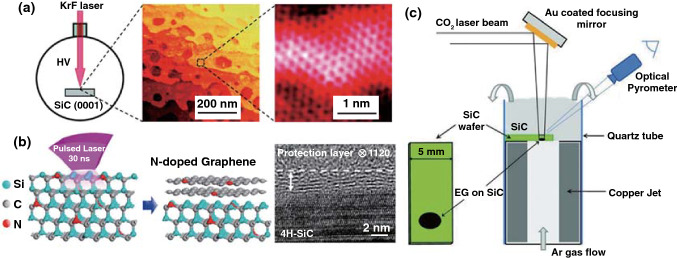
**a** Illustration of laser-induced epitaxial graphene synthesis [49]. **b** Schematic illustration of the synthesis method for laser-induced N-doped graphene on N-doped SiC substrate and cross-sectional HRTEM image of multilayer N-doped graphene on 4H-SiC (0001) [50]. **c** Schematic diagram of the CO_2_-laser-induced epitaxial growth of graphene on SiC wafers [51]

 As mentioned above, although the laser-processed rGO had good electrical conductivity, abundant defects remain existed in the *sp*^2^ carbon network. As a practical method, the chemical vapor deposition (CVD) and epitaxial growth (EG) of graphene were feasible methods to produce large-scale graphene with high quality, while the fabrication of graphene patterns through CVD or EG was commonly time consuming and costly. Thus, developing a rapid approach for fabrication of graphene is essential. Lu et al. [46, 47] developed a fast growth method of graphene by laser direct writing (a focused continuous-wave laser beam with *λ* = 532 nm, laser power = 5 W) on a thin Ni foil in a CH_4_ and H_2_ atmosphere, which can precisely control the position of graphene pattern and realize the improvement of growth rate than that of general CVD method. For instance, a graphene pattern with an area of 10 × 10 μm^2^ can be rapidly grown in 0.2 s. A similar strategy was also reported by Zhong et al. [48]. Besides the CVD method, epitaxial growth of graphene was another choice. In order to solve the problem of high growth temperature for EG method, Salleo et al. [49] demonstrated a scalable epitaxial graphene synthesis technique based on laser-induced surface decomposition of the Si-rich face on silicon carbide (SiC) single-crystal (pulsed KrF laser with 248 nm and 25 ns) (Fig. 3a). Furthermore, Choi and Lee et al. [50] reported epitaxial growth of solid-phase N-doped graphene on the doped silicon carbide (SiC) substrate driven by pulsed XeCl excimer laser (*λ* = 308 nm, pulse duration ∼ 30 ns). Compared with the gas-phase doped graphene method, this strategy can precisely control the doping concentration of graphene by tuning the dopant concentration of SiC substrate (Fig. 3b). The above reported laser epitaxial growth of graphene was all performed in a high-vacuum condition. Yannopoulos and Siokou et al. reported that epitaxial growth of large-area graphene on the surface of SiC(0001) using a continuous-wave infrared CO_2_ laser was realized (Fig. 3c) [51], which does not require high-vacuum or strict sample-chamber conditions. In addition, another advantage for the CO_2_ laser was manifested that the very high heating rate derived from CO_2_ laser efficiently avoided the different Si desorption rates from adjacent SiC steps to form homogeneous graphene. In conclusion, the laser synthesis technique by means of EG presented many advantages. Firstly, large-scale production, time and cost saving as well as patterning without nanolithography were still exhibited in laser processing. Secondly, this method operates at low temperatures on account of the laser-induced local high temperature, which does not necessitate high vacuum and/or SiC pre-treatment. Lastly, controllable doping concentration of graphene can be easily realized by tuning the dopant concentration of the SiC substrate.

#### Laser Synthesis of Diamond-like Carbon

**Fig. 4:**
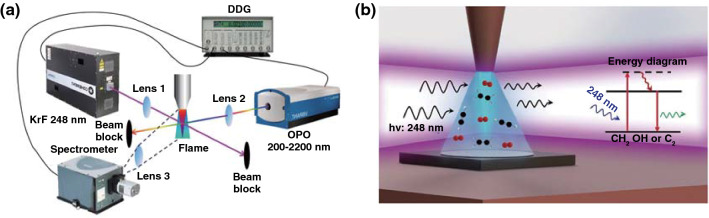
**a** Schematic diagram of the optical emission spectroscopy (OES) and laser-induced fluorescence (LIF) setup to characterize the species in the combustion flame with UV laser irradiation. **b** Schematic illustration of the UV-laser-assisted diamond combustion CVD setup [53]

Graphene has good electrical conductivity and transparency to some extent. Diamond-like carbon (DLC) as another amorphous form of carbon exhibited the electrical insulator property due to kinship with diamond and visible and infrared range transparency. Therefore, combining the properties of DLC and graphene could innovatively obtain a transparent conductive material coating on an insulated transparent substrate. Laser synthesis of diamond-like carbon and graphene provided an accurate, convenient, clipping method to realize this innovative solution. Stock et al. [52] explored the preparation of DLC thin films from a high-purity graphite target by pulsed laser deposition (PLD). It is worth noting that the high-vacuum condition with residual pressure less than 10^−8^ mbar was essential. Then by using the obtained DLC as substrate, UV laser surface annealing was performed to modify the first atomic layers of DLC thin-film structure by graphene-like layers with high conductivity. It demonstrated that the obtained graphene layer on DLC had comparable conductivity and transparency performances to those of ITO. In addition, Fan et al. [53] used hydrocarbon species including a mixture of acetylene (C_2_H_2_), ethylene (C_2_H_4_) and oxygen (O_2_) as the precursor, a combustion torch was used to produce the flames and an ultraviolet (UV) laser was used to excite the combustion species in the direction of perpendicular to the combustion flames and parallel to the substrate (Fig. 4a, b). By analyzing the nucleation process of diamond growth, they proposed the two critical surface reactions during diamond growth: (1) addition of reactive hydrocarbon radicals to the active surface sites and (2) H-abstraction from hydrocarbon radicals to create more reactive sites to accept hydrocarbons and stable *sp*^3^-hybridized carbon bonds. Therefore, the H-abstraction from hydrocarbons by UV laser-induced photolysis to produce abundant reactive species play a dominant role in promoting diamond growth, which can shorten the diamond nucleation time and suppressed the nondiamond carbon accumulation.

#### Laser Synthesis of Heteroatom-Doped Carbon

During the laser processing, the high localized temperature caused by the photothermal effects of laser played a key role in material preparation, meaning that laser was used as a heat source. So ordinary materials prepared by heating synthesis can be similarly achieved by laser. Therefore, heteroatom-doped carbon can also be rapidly synthesized by laser and realize patterning in a large scale. In terms of laser-induced-doped graphene, Professor Tour’s group prepared porous B-doped graphene with promising electrochemical performance by introducing H_3_BO_3_ into PI, as mentioned in Sect. 2.1.1.2 [35]. Alshareef et al. [54] prepared nitrogen-atom-doped graphene using urea-containing polyimide as the precursor on Cu foil. The binder-free, additive-free, and conductive anodes for Na-ion battery was fabricated by single-step laser-based transformation. Li et al. [55] prepared the S- and N-doped graphene patterns on glass and polyethylene terephthalate (PET) substrates by using organic polybenzimidazole (PBI) ink as the precursor. During the fabrication process, a UV laser beam with 355 nm wavelength was used to break the C–S bond (2.8 eV) from dimethyl sulfoxide (DMSO) and the C–N bond (3.14 eV) from polybenzimidazole. In addition, Professor Yuge prepared B- and N-codoped single-walled carbon nanohorns by CO_2_ laser processing of a boron-containing carbon target under nitrogen atmosphere [56]. Besides of nitrogen, other heteroatom-doped graphene was also synthesized by laser processing. Ruoff et al. [57] reported a Raman laser (*λ*_laser_ = 488 nm) irradiation to prepare the F-doped graphene with fluoropolymer-covered graphene as the precursors. The laser-induced decomposition of the fluoropolymer produced active fluorine radicals and then reacts with the *sp*^2^-hybrized graphene to form C–F bonds. Therefore, similar to laser synthesis of graphene and other carbon materials, introduction of heteroatom precursors into the raw materials was essential for laser synthesis of heteroatom-doped carbon. By considering the light-absorption characteristics of the carbonization precursor and the precision of the laser, the appropriate type of laser can be selected, and then, heteroatom-doped carbon materials were prepared through regulating parameters such as laser power, frequency, and pulse width of laser.

As mentioned above, laser synthesis of carbon materials from polymers is mainly based on the absorption of laser by the precursor, which results in the heating effects. As the consequence, the CO_2_ laser is the preferable choice because that the infrared laser with a long wavelength is conducive to the photothermal reaction to carbonization. For the laser-induced rGO from graphene oxides, although the photothermal effect and photochemical effect simultaneously happened, photochemical effect through rebinding of atoms within GO induced by laser was dominated. Thus, laser wavelength covering UV, Vis and NIR wavelength range is all adaptable. The effects of parameters of laser on the composition nature of obtained carbon materials are summarized in Table 1.

**Table 1:** Effects of parameters of laser on the composition nature of obtained carbon nanomaterials

Precursor	Laser source	Parameters	Properties of carbon	Applications	References
GO	Nd-YAG laser	1064 nm, ms	rGO with low resistance		[21]
GO		355 nm, nm	rGO with expanded structure	Micro-supercapacitors	[22]
GO fibers	Argon-ion laser	458 nm	rGO with high conductivity	Moisture-sensitive fiber actuator	[23]
GO aerogel		1 W, ms	Heteroatom-doped rGO bulk	Eletrocatalysis, Li-ion batteries, supercapacitor, methanol oxidation	[24]
GO	LightScribe DVD optical drive	Infrared laser	rGO sheets	Electrochemical capacitors electrode	[25]
GO	LightScribe DVD burner	Infrared laser	rGO sheets	Micro-supercapacitors	[26]
Commercial polymer films	CO_2_ laser	10.6 μm, 14 μs, 2.4–5.4 W	Porous graphene films (only PI and poly(etherimide) can form)	Micro-supercapacitors	[32]
Polyimide (PI)	CO_2_ laser (75 W)	10.6 μm	Porous graphene	Gas sensors	[34]
Boric acid containing PI	CO_2_ laser	10.6 μm, 14 μs, 4.8 W	Porous graphene	Micro-supercapacitors	[35]
PI, laminated object manufacturing	CO_2_ laser (75 W)	10.6 μm	Graphene foams	Energy storage and stress sensor device	[37]
Ni/sucrose layers	CO_2_ laser (75 W)	10.6 μm	Graphene foams	Damping materials	[38]
Cloth, paper or food	CO_2_ laser (75 W) CO_2_ laser (50 W)	10.6 μm 9.3 μm	Porous graphene	Micro-supercapacitors	[33]
Teflon	CO_2_ laser (50 W)	9.3 μm, 5.0 W	Fluorinated carbon materials	Tuning the band gap of FG	[39]
PI paper	CO_2_ laser	10.6 μm, 0.75–2.0 W	Graphene paper	Strain sensors, micro-supercapacitors, superhydrophobic membrane, acetone sensor, antibacterium device	[41]
CH_4_ and H_2_	Solid state laser	532 nm, continuous wave, 5 W	Graphene patterns		[46–48]
SiC single crystal	KrF laser	248 nm, 25 ns, 1.2 J cm^−2^	Epitaxial graphene on SiC		[49]
High doped 4H-SiC wafers	XeCl laser	308 nm, 30 ns	N-doped epitaxial graphene		[50]
6H-SiC	CO_2_ laser	10.6 μm, continuous wave	Epitaxial graphene on SiC		[51]
Graphite target	KrF laser	248 nm, 25 ns, 600 mJ	Diamond-like carbon	Optical and photovoltaic applications	[52]
Urea-doped PI film	CO_2_ laser (75 W)	10.6 μm, 14 μs, 2.4–5.4 W	N-doped graphene	Na-ion batteries	[54]
Polybenzimidazole (PBI) in DMSO	UV laser	355 nm, ps	N, S-doped graphene	Flexible electronics, imbedded sensors, smart wearables, and lithium-ion batteries	[55]
Boron-containing carbon target	CO_2_ laser	Continuous wave, 3.5 kW	B, N-doped single-walled carbon nanohorns		[56]
Fluoropolymer-covered graphene	Raman laser	488 nm	F-doped graphene		[57]

### Non-carbon Nanomaterials

#### Laser Synthesis of Non-carbon Nanomaterials in Non-aqueous Environment

Except for the synthesis of different carbon nanomaterials, laser processing was also commonly developed to fabricate some other non-carbon nanomaterials, such as metal oxides, metal carbides and metal disulfides, especially as a large-scale and fast fabrication method. Laser synthesis of non-carbon nanomaterials is primarily based on the photothermal effects and/or the photochemical effects induced by the irradiated laser, where the absorption of laser by the precursors results in some specific effects. Specially, ablation or heating dominate the nanomaterials formation during the laser synthesis of non-carbon nanomaterials in non-aqueous environment.

##### Metal and Metal Oxides

 Metal nanomaterials and metal oxide nanomaterials have been widely used in various applications in energy storage [58], energy conversion [59] and environmental treatment [60]. In recent years, many synthetic methods, including chemical vapor deposition (CVD) [61], liquid chemical method [62–65], hydrothermal method [66] and electrochemical method [67], have been developed. However, large-scale manufacturing of these nanocrystals with high performance remained very difficult. Therefore, the laser synthesis method attracted increasing attention for its fast, facile and large-scale fabrication process, as well as operation at low temperatures [68].

**Fig. 5:**
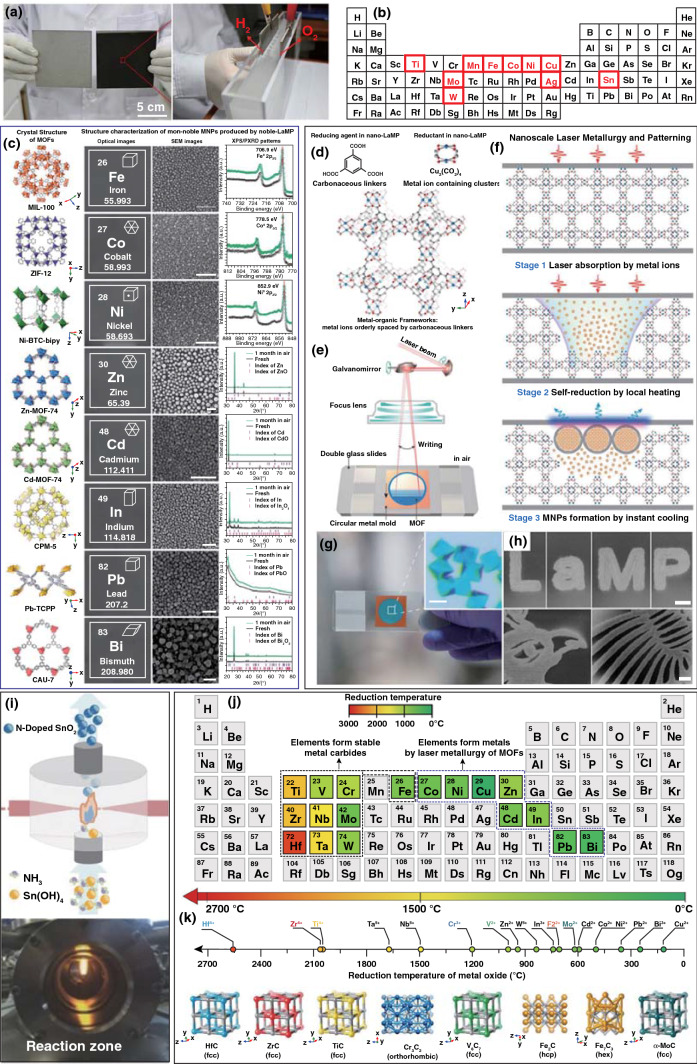
**a** Digital photograph of Ni plates (10 × 10 cm^2^) before (left) and after (right) laser ablation and the overall water splitting device by Ni plate. **b** The applied metals and alloys highlighted within a periodic table [69]. **c** Various non-noble MNPs produced by the nano-LaMP method in air displayed along with their corresponding MOF crystals. From left to right, the crystal structure of MOFs, optical images, SEM images, PXRD patterns, and XPS patterns of MNPs. HKSUT-1 precursor **d** and illustrations of the experimental setup **e** for the nano-LaMP method. **f** Mechanism for the production of MNPs by laser irradiation on MOFs in air. **g** Optical image of the MOF crystals prepared for nano-LaMP. The scale bars are inset in **g** 20 μm and **h** 200 μm [77]. **i** Illustration of the laser pyrolysis process of N-doped SnO_2_ [74]. **j** Periodic table shows the reduction temperatures of various metallic elements according to Ellingham diagram. **k** TMCs synthesized in this study using MOF as precursor and laser as energy source [78]

As mentioned above, ablation or heating dominated the nanomaterials formation during the laser synthesis of non-carbon nanomaterials in non-aqueous environment. With regards to the laser ablation synthesis of nanomaterials, a target surface was irradiated by a beam of high-energy pulsed laser. The reflection and absorption simultaneously occurred, and once the absorbed laser energy exceeded the evaporation temperature of the target, the target would melt and evaporate a large number of atoms, electrons and ions, thus forming a plasma on the surface of the target. After shifting the pulsed laser, the plasma expanded and then cooled and crystallized to prepare nanomaterials. For instance, Li et al. [69] reported a general strategy to manufacture a series of metal oxides (MO_*x*_, M = Ti, Mn, Fe, Co, Ni, Cu, Mo, Ag, Sn, W, and NiFe) with hierarchical nanostructure on corresponding metal substrates by laser ablation. When using as the electrocatalysts, additional binders were avoided to assist the adhesion of electrocatalyst nanoparticles on substrates. The universality for various metal oxides synthesis, large-scale manufacturing and fast synthesis process demonstrated the advantages of laser ablation synthesis (Fig. 5a, b). Zheng et al. [70] grew preferentially [001]-oriented BiVO_4_ on FTO substrates through the laser ablation of the BiVO_4_ target. Compared with the BiVO_4_ with randomly oriented grains produced by sol–gel drop-casting method, the laser ablation method has a lot of advantages in sample preparation. This system is capable of controlling the variables during the synthesis such as temperature, type of reactive gas and the pressure; thus, epitaxial films as well as various morphologies, compositions and phases of products can be fabricated. As demonstrated, this preferentially [001]-oriented BiVO_4_ exhibited higher performance as the photoanode materials for photoelectrochemical water splitting. Most of the laser ablation synthesis strategies of metal and metal oxides were utilized in the liquid environment, which was discussed in Sect. 2.2.2.

With regards to the laser heating synthesis of nanomaterials, the significantly localized and rapid photothermal effect induced by a focused laser was employed to replace the conventional thermal treatment for the nucleation and growth of nanomaterials. For instance, the laser-assisted pyrolysis method was a traditional synthesis strategy for metal oxide nanomaterials [71–73]. Xu et al. [74] fabricated N-doped SnO_2_ nanoparticles through this laser-assisted pyrolysis process, which using Sn(OH)_4_ suspension as the precursor with NH_3_ for doping and a gaseous sensitizer (C_2_H_4_) for absorbing laser photons (Fig. 5i). The as-synthesized N-doped SnO_2_ nanoparticles exhibit excellent cycling stability and rate capability as anode materials in lithium-ion batteries (LIBs), which was attributed to the formed unreactive Sn–N bonding in the structure during charge/discharge and the small particle sizes as a result of laser pyrolysis. However, the heat transfer of the laser-assisted pyrolysis process was usually limited by the gaseous precursor and generally obtained the product with poor phase purity. Metal–organic frameworks (MOFs) provided ideal contact between carbonaceous and metallic precursors on an atomic scale and preserved efficient photothermal conversion under laser irradiation [75]. Ogale et al. [76] selected MOFs of zeolitic imidazolate frameworks (ZIF-67) as precursors to achieve the metal and heteroatom-doped, porous graphene hybrid electrodes by laser direct writing, which set a precedent for laser carbonization of MOF under ambient for micro-supercapacitor application. Deng et al. [77] selected different porous MOFs to be processed by pulsed laser, a series of metal nanoparticles including Fe, Ni, Co, Cu, Cd, Zn, In, Pb, and Bi with uniform sizes and gaps were produced (Fig. 5c). The self-reduction process of metal ions atomically dispersed in MOFs and then assembled across the pores under laser irradiation, which contributed to the formation of metal nanoparticles (MNPs). In addition, the formed thin layers of graphene on the surface can efficiently protect metal nanoparticles from oxidation and aggregation. By controlling the processing position of laser, the conversion sites from MOFs to MNPs were controlled and the desirable patterns were finally generated (Fig. 5d–h). Thus, this method can be called nanoscale laser metallurgy and patterning (nano-LaMP). The mechanism of the MNPs production through laser irradiation on MOFs was proposed and then confirmed in this article (Fig. 5f). Firstly, the coordination between metal ions and organic ligands in MOFs leads to high light absorptivity. Secondly, the high photothermal conversion efficiency leads to the rapidly increased temperature of the MOFs, and the increased temperature was high enough to decompose the organic ligands in the MOF. Then, a local reductive atmosphere composed of H_2_, CH_4_, CO, and C_2_H_2_ was created to reduce the metal cations in MOF and subsequently assembled to form MNPs with protective graphene layers under the synergy effect of the reductive atmosphere and extremely high temperature at the local position resulted from laser movement. This work provided a new perspective for laser synthesis of nanopowders free of prefabrication of films. By using the same nano-LaMP method, Deng et al. [78] recently found out that metal species in the MOF resulted in different final products after nano-LaMP. For instance, the metal featuring with unsaturated d orbitals [Hf(5d^2^), Zr(4d^2^), Ti(3d^2^), Cr(3d^5^), V(3d^3^), Fe(3d^6^) and Mo(4d^5^)] (Fig. 5j) in MOF was inclined to convert to transition metal carbide nanoparticles (TMCs), because that these metals could form hybrid orbitals with carbon to form metal carbides rather than metal nanoparticles. In addition, these TMCs needed an extremely high temperature to form stable metal carbides compared with the formation of metals (Fig. 5j); therefore, the extremely high temperature resulted from pulsed laser made it possible for the reduction of metal ions to TMCs. In conclusion, the characteristic of metal species in MOFs as well as the laser synthesis method along with high temperature, reductive atmosphere (CO and H_2_) and instant heating and cooling facilitated the formation of a large variety of TMCs (Fig. 5k).

Hybrid materials composed of graphene and metal oxides were also developed to be fabricated by laser processing, as laser-induced graphene from polymers has been widely studied and significant advances have been made. Tour et al. [79] scribed a metal-complex containing polyimide film by the CO_2_ laser, different metal oxide nanoparticles including Co_3_O_4_, MoO_2_, and Fe_3_O_4_ embedded in porous graphene were formed. This work provided an advanced method for synthesis of metal oxide nanoparticles/graphene hybrid materials by laser processing. Meanwhile, through two-step laser scribing, Co_3_O_4_/LIG, MnO_*x*_/LIG and NiFeO_*x*_/LIG were also prepared by this group [80], where the first laser-induced graphene and then soaking the LIG by metal ions solution for second-time laser processing were contained. In addition, they verified that the nanoparticles could convert from metal oxides to metal dichalcogenides, but the lateral sulfating treatment was essential.

##### Metal Carbides

**Fig. 6:**
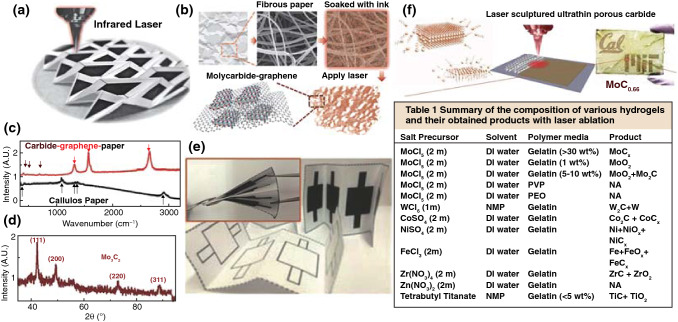
**a** An optical photograph of a fabricated origami structure after the direct laser-write MCG patterning process (black color areas) on a paper substrate (white color areas). **b** Schematic illustration of the simplified MCG process from fibrous paper, soaked with the gelatin-mediated ink containing Mo^5+^ ions, laser conversion process, to the resulting MCG composites. **c** Raman spectroscopy and **d** XRD patterns of the MCG sample. **e** Two partially folded, four 2 × 2 cm^2^ electrodes on a paper substrate: (top) after the laser conversion process and (bottom) before the conversion process. (inset) A fully folded device with two electrodes on top and two electrodes at the bottom for a two-capacitor in sandwich structure to be connected in series or parallel as supercapacitors [81]. **f** IR laser ablation generates highly porous structures and the summary of the composition of various hydrogels and their obtained products with laser ablation [82]

As laser scribing is a relatively common method for synthesis of carbon materials, the metal carbide-graphene composites synthesized by laser were also developed. Lin et al. [81] developed a laser direct-write patterning process to fabricate molybdenum carbide–graphene (MCG) conductive composites directly on paper substrates, which was realized by soaking the fibrous paper in the Mo-gelatin ink and then direct laser conversion of MCG by a CO_2_ laser with wavelength of 10.6 μm (Fig. 6a, b, e). After the conversion, the coexistence of graphene and Mo_3_C_2_ was verified by Raman and XRD results (Fig. 6c, d). It was demonstrated that gelatin-based ink could produce higher conductivity than other hydrogel polymers, which was attributed to the formation of lamellar morphology in layer-by-layer form and the absorption enhancement of CO_2_ laser by strongly binding with Mo^5+^ ions under the effect of porcine skin peptides with rich amino acid functional groups. This work potentially promoted laser synthesis of metal carbide-graphene composites for practical applications in recyclable and disposable paper-based electronics. Based on this work, this group further proposed the ultrathin carbides on versatile substrates through CO_2_ laser processing of a lamellar hydrogel/metal-ion matrix [82]. Different metal ions were investigated, and it can be observed that Co^2+^ and W^6+^ gelatin produced the composites of carbides and metal hybrid phases. The Ti^4+^ and Zr^4+^ gelatin hydrogels were converted to carbide and oxide composites, while Fe^3+^ and Ni^2+^ gelatin dominantly produced metal oxides with small portions of carbides. No crystalline phase was produced by Zn^2+^ gelatin (Table in Fig. 6f). Therefore, the finally formed products are determined by two critical factors, including the efficiency of laser absorption by the metal-gelatin and the activation energy of carbonization, which determine whether the carbides can be formed.

##### Metal Disulfides

**Fig. 7:**
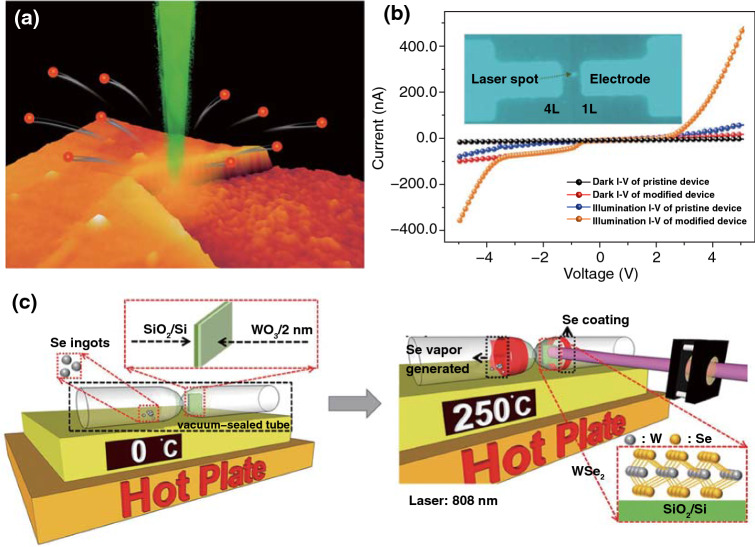
**a** Schematic of controllably thinning multilayered MoS_2_ down to single layer by laser [84]. **b** Typical I–V characteristics of pristine and modified photodetection devices at dark and laser illumination condition [85]. **c** Schematic illustration of the rapid synthesis of the WSe_2_ by the LIAS process [90]

In the past decades, numerous attempts to fabricate the transition metal dichalcogenides (TMDs) with monolayer by the chemical vapor deposition (CVD) process had been performed [83]. However, on account of being time-consuming, low thermal efficiency and required transfer of the CVD process, developing alternative synthesis methods were required. Laser synthesis of the transfer-free TMDs provided a promising strategy. In terms of laser synthesis of TMDs nanomaterials, laser thinning and laser annealing were two commonly used strategies. The laser thinning method relies on the sublimation of the outermost layer and heat is introduced by the absorption of laser. Because that the van der Waals force between TMDs layers is very weak, the produced heat is difficult to transfer through the substrate; thus, the sublimation of the outermost layer occurs with the bottom layer of TMDs which remain tightly attaching on the substrate. For example, Steele et al. [84] employed a focused laser beam to fabricate monolayer MoS_2_ from multilayered MoS_2_ flakes by the laser-thinning method (Fig. 7a). A MoS_2_ monolayer domain surrounded by multilayer film through the growth of MoS_2_ films and subsequent laser thinning process was also produced by Sow et al. [85]. With this approach, functional junctions with different band gaps were readily fabricated by laser thinning MoS_2_ domains with different thicknesses, which exhibited superior photoresponse characteristics (Fig. 7b). In addition, laser ablation-induced defects on TMDs also depends on the sublimation of part of the TMDs through the photothermal effects by laser. In this case, a series of van der Waals heterostructure arrays, including VSe_2_/WSe_2_, NiTe_2_/WSe_2_, CoTe_2_/WSe_2_, NbTe_2_/WSe_2_, VS_2_/WSe_2_, VSe_2_/MoS_2_, and VSe_2_/WS_2_, were prepared by laser-patterning periodic arrays of nucleation sites on monolayer or bilayer semiconducting TMDs to selectively grow metallic TMDs. The laser ablation for patterning played an important role in providing nucleation sites for growth of TMDs [86].

The laser annealing method is using laser as a heating source to promote the synthesis reaction between different precursor species. For instance, Tenne et al. [87] creatively used ultrashort-pulse laser to process a mixture of bulk transition-metal dichalcogenides and Pb/PbO to fabricate inorganic fullerene-like and nanotube-like TMDs nanostructures, the catalytic effects of metal assisted with the ultrafast laser-induced thermal annealing contributed to the formation of different TMDs nanostructures. Lamberti et al. [88] reported rapid one-pot synthesis of MoS_2_-decorated laser-induced graphene (MoS_2_-LIG) by direct writing of polyimide foils coating with a layer of MoS_2_ dispersion. After laser writing, the MoS_2_ can be in situ decorated into the surface of 3D arrangement of agglomerated and wrinkled graphene flakes. The metal sulfide (CdS or PdS) decorated graphene composites by laser-induced synchronous carbonization and sulfidation of a metal-complex-containing polyethersulfone layer were also reported [89]. The prepared LI-CdS/PdS-G@ITO afforded both the porous structure and uniform distribution of metal sulfides, which presented an efficient photoanode for photoelectrochemical detection of Cu^2+^ with significant selectivity and sensitivity. WO_3_ and Se powders can also be used as the reaction precursors, Chueh et al. [90] utilized laser annealing as a heating source to synthesize few-layer WSe_2_ by the reaction between WO_3_ and Se gas (Fig. 7c). The phase transformation from WO_3_ to WSe_2_ was confirmed by Raman spectrum, and the distinct interface between WO_3_/WSe_2_ and SiO_2_ can be clearly observed in the optical images of the deposited film before and after laser annealing. This laser irradiation-assisted selenization (LIAS) process not only had the advantages of being ultrafast (< 20 min), low-cost and low synthesis temperature, but also exempted from the additional transfer process of TMDs. Furthermore, the laser irradiation-assisted synthesis process can also be adapted to the other TMDs such as MoSe_2_, which demonstrated wide applicability. It is noted that most of the fabrication process of transition metal dichalcogenides by laser is based on two-step treatment, including the first growth of TMDs film and then laser treatment; the LIAS method provides a new strategy to synthesize TMDs by the gas–solid reaction utilizing laser as heating source.

##### Heteroatom-Doped Transition Metal Compounds

Except for the laser synthesis of heteroatom-doped carbon, the laser processing technique was also utilized for incorporation of heteroatoms into the transition metal compounds. For instance, the laser-induced chemical modification of suspended TMD monolayer films via local exchange of the chalcogen atoms has been reported [83]. With the proposed method, total or partial locally replacing selenium by sulfur atoms was achieved, which exhibited an excellent performance in nanodevices composed of heterogeneous 2D materials. In addition, the synthesis of oxide thermoelectric material of Bi_2_Sr_2_Co_1.8_O_*x*_ via laser floating zone [91] and the synthesis of rare-earth-doped SrTiO_3_(Y, Dy, Sm, Pr, La-doped SiTiO_3_) via laser melting [92] also have been reported, demonstrating the potential advantage of laser synthesis of heteroatom-doped transition metal compounds. Liu et al. [93] used the laser-drilled micropores strategy to prepare the hybrid electrocatalyst of Fe-doped NiS_2_/MoS_2_ on a CNT film with periodic micropores, the hybrid electrocatalyst provided strengthened interfacial interactions and abundant active sites, improving both thermodynamic catalytic activity and active site density. Therefore, different laser synthesis strategies can be utilized for incorporation of heteroatoms into transition metal compounds, the optimization of laser power, exposure time, and reactive atmosphere is crucial to control the heteroatoms dopant on the transition metal compounds.

##### Other Non-carbon Nanomaterials

Organic–inorganic halide perovskites have emerged as promising materials for optoelectronics, especially photovoltaics [94, 95]. As well known, the optimum annealing crystallization temperature for MAPbI_3_ perovskite film was 100 °C. However, it is limited by the energy and time consuming, and incompatibility with low temperature fabrication requirement of flexible polymers or plastic substrates for the traditional thermal-annealing process, laser annealing-induced crystallization process provided a novel annealing approach for precisely controlling the crystallization position and achieving larger grains with lower density of trap states and higher carrier mobility, especially for reducing carrier recombination and enhancing photovoltaic performance of perovskite solar cells (PSCs). Yu et al. [96] introduced a continuous-wave laser (*λ* = 450 nm) irradiation to rapidly crystallize and prepare CH_3_NH_3_PbI_3_ films with dense and homogeneous grains. The obtained planar-heterojunction solar cells showed the optimal efficiency of 17.8% with a remarkably high open-circuit voltage of 1.146 V. In addition, Kim et al. [97] used the near-infrared (NIR) laser (*λ* = 1064 nm) to introduce controllable crystallization of MAPbI_3_ perovskite solar cells, the inverted-type perovskite solar cells with efficiency of 11.3 and 8.0% on typical glass and flexible polymer substrates, respectively, were demonstrated. Recently, Yan et al. [98] also used the fast laser annealing to induce a higher temperature and selectively grew large perovskite grains. By tuning the laser processing parameters, including the scanning speed, laser wavelength and output power, the optimum perovskite films with high crystallinity and perovskite solar cells with high energy conversion efficiency and durability were successfully fabricated. Besides the crystallization of perovskite films for PSCs, the fs laser processing Bi_2_WO_6_ with a strong enhancement of the structural organization and crystallinity were also reported [99].

Different lasers commonly induced different temperatures due to a different absorption capability of the precursor, thus produced different types of non-carbon materials. In conclusion, the characteristics of precursor species and the laser radiation (laser intensity, wavelength, and interaction time, etc.) are of vital importance. The effects of precursors and parameters of laser on the composition nature of the obtained non-carbon materials are summarized in Table 2.

**Table 2:** Effects of precursors and parameters of laser on the composition nature of the obtained non-carbon nanomaterials

Precursor	Laser source	Parameters	Products	Applications	Refs
Metal substrates (Ti, Mn, Fe, Co, Ni, Cu, Mo, Ag, Sn, W, and NiFe)	Nanosecond (ns) pulsed laser	532 nm, 12 ns, 0.4 mJ	Hierarchical nanostructured metal oxides	Electrocatalytic water splitting	[69]
SiH_4_, C_2_H_4_	CO_2_ laser	10.6 μm, 18 kHz	Si@C	Anode material for lithium-ion batteries	[71]
Toluene or pyridine, SF_6_	CO_2_ laser	10.6 μm	Undoped or N-doped carbon nanodots	Visible-light photocatalysis	[72]
Sn(oh)_4_, NH_3_, C_2_H_4_	CO_2_ laser	10.6 μm	N-doped SnO_2_	Anode material for lithium-ion batteries	[74]
MOFs	Nanosecond pulsed laser	1.5 W	Non-noble metal nanoparticles	SERS devices	[77]
MOFs	Nanosecond pulsed fiber laser	1064 nm, 80 ns, 20 kHz, 6 W	Transition metal carbides	Catalytic CO conversion	[78]
Metal-complex containing polyimide (MC-PI) film	CO_2_ laser	10.6 μm, 14 μs, 4.8 W	Porous graphene with embedded metal oxide nanocrystals	ORR	[79]
PI, Co(NO_3_)_2_	CO_2_ laser	10.6 μm, two-step laser processing	Co_3_O_4_/LIG	Zn-air and Li-O_2_ batteries	[80]
PI, MoS_2_ dispersion	CO_2_ laser	10.6 μm	MoS_2_-decorated LIG	Flexible supercapacitor	[88]
PI, ZIF-67	CO_2_ laser	10.6 μm, 9–10 W	Metal-decorated and heteroatom-doped porous graphene	Micro-supercapacitors	[76]
Multilayered MoS_2_ film		532 nm, 100 mW cm^−2^	MoS_2_ single layer	Photodetector device	[84, 85]
Mixture of MoS_2_ (or WS_2_) and Pd	Nd:KGW laser	513 nm, 170 fs,	WS_2_ or MoS_2_ nanostructures		[87]
WO_3_ film, Se ingots		808 nm, continuous wave	Few-layer WSe_2_	Field effect transistor (FET) devices	[90]
WSe_2_, H_2_S	Diode laser	532 nm, 0.3 mW	S-doped WSe_2_		[83]
Fibrous paper, gelatin inks containing Mo^5+^	CO_2_ laser	10.6 μm, 0.5–4.0 W,	Mo_3_C_2_-graphene composites	Electrochemical sensors, piezoelectret generator, supercapacitors	[81]
Gelatin containing Mo^5+^, W^6+^, Co^2+^	CO_2_ laser	10.6 μm, 2 W	MoC_x_, WC_x_, CoC_x_ on versatile substrate	Flexible supercapacitor, solar-energy harvesting membrane	[82]
MAPbI_3_ perovskite film	Continuous-wave laser	1064 or 450 or 405 or 660 nm	Crystallization of MAPbI_3_ perovskite film	Perovskite solar cells	[96–98]
Bi_2_WO_6_	Ti:sapphire laser	800 nm, 30 fs, 200 mW	Crystallization of Bi_2_WO_6_		[99]

As mentioned above, laser as a synthetic technique can construct various nanostructure during the laser processing. However, more nanostructure styles have not been realized compared with those constructed by wet chemical methods. Therefore, in situ transformation from the preconstructed nanostructures through the thermal effect of the unfocused laser is also a potential proposal for expanding the application of laser synthesis process. Furthermore, laser as a synthetic technique is not limited to the aforementioned applications. Laser synthesis have great potential for many other development directions. For instance, more compound species can be controllably synthesized by laser ablation under different atmospheres instead of only air or Ar atmosphere, such as the synthesis of nitrides under the N_2_ or NH_3_ atmosphere, synthesis of sulfides under the H_2_S atmosphere, synthesis of carbides under the CH_4_ atmosphere and even synthesis under the CO_2_ atmosphere for oxides or carbides with unique properties.

#### Laser Synthesis of Nanomaterials in Liquid

The laser synthesis of nanomaterials can be performed in two distinct conditions, including the non-aqueous environment and the liquid environment. Therein, laser synthesis of nanomaterials in liquid environment has been widely applied as a versatile technique to construct various colloidal nanostructures. According to the processing mode and the formation mechanism of nanoparticles, laser synthesis can be classified into three methodologies: laser ablation in liquids (LAL), laser fragmentation in liquids (LFL), and laser melting in liquids (LML) [100]. As a synthesis technique of nanomaterials, LAL and LFL were the most commonly utilized methods to prepare nanomaterials. Then in this section, we reviewed on the recently researched nanomaterials with innovative phases through LAL and LFL in liquid.

##### Laser Synthesis of Nanomaterials by Laser Ablation in Liquid

 The LAL technique has inspired extensive researches on nanomaterial synthesis and modification in liquid as well as the properties and applications of the LAL-induced nanomaterials. Two LAL techniques, including the “top-down” synthesis of pulsed laser ablation of a solid target in liquids (PLAL) and the “bottom-up” method of pulsed laser irradiation of colloidal nanoparticles in liquids (PLICN) were promising liquid-based laser protocols for complex nanomaterials. Most PLAL synthesis processes were realized by laser heating the solid target, and then, the initial products including plasma, vapor, and droplets with micro-nanosizes could be produced, which further reacted with the liquid solvent to form the final colloidal nanoparticles [101]. The PLICN technique was that pulsed laser irradiation of a precursor solution, where nucleation and growth of nanomaterials occurred as a result of the laser-induced chemical reactions [102].

As mentioned above, the PLAL process went through the plasma and the gasification stage, and the subsequent substance diffusion and interaction with liquid solvents after bubble collapse. In general, lasers with a short pulse-width and high-energy density were beneficial to the generation of plasma and vapor products, while nanodroplet products were usually caused by lasers with low-power density. The formation of nanostructures was attributed to their interaction with the surrounding liquid solvents and the ultrafast quenching of laser. Owing to the variety of solid targets and liquid solvents, nanomaterials including alloys, metals, oxides, hydroxides, nitrides, carbides, sulfides and composites were achievable by PLAL [103].

**Fig. 8:**
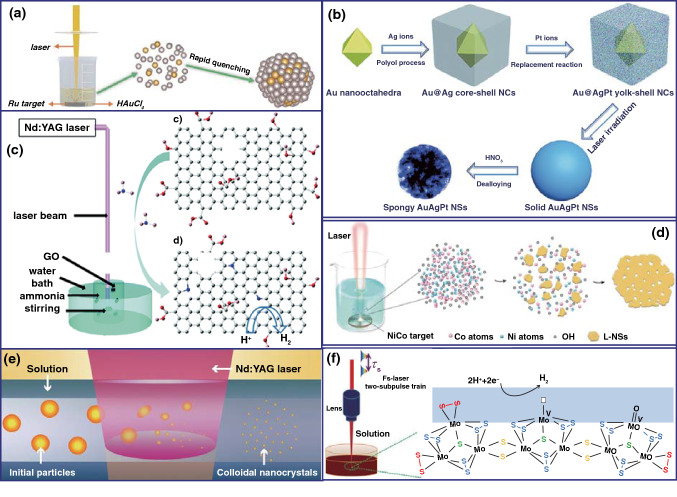
**a** Schematic setup of PLAL and the formation process of RuAu SAA nanoparticles [111]. **b** Schematic diagram for the formation process of spongy AuAgPt [113]. **c** Illustration of the preparation of nitrogen-doped graphene oxide by laser irradiating a solution containing graphene oxide and ammonia [115]. **d** Laser ablation of CoNi alloy target in 1 M KOH solution [119]. **e** Schematic illustration of the fabrication processes of laser synthesis and processing of colloids [127]. **f** Controllable synthesis of nanosized amorphous MoS_x_ by fs laser [132]

In terms of the synthesis of metals and alloys by PLAL, the “bare” unprotected Au nanoparticles were frequently prepared by laser ablation of Au target or Au particles in solution [104–106], which were applied for the catalytic applications. In order to enhance the catalytic activity of individual gold nanoparticles (AuNPs), using AuFe alloys plate [107] or Pt-Au powder-mixture compression [108] as the target for PLAL synthesis method, a homogeneous Au-Fe nanoalloy with a Fe content up to 11 at% for OER and PtAu alloys for the 4-NP reduction were achieved. Replacing the deionized water by some metal ions solution, the PLAL technique can also be utilized to synthesize alloys. Mukherjee et al. [109] proposed a laser ablation of Co target in K_2_PtCl_4_ solution, the PtCo nanoalloy embedded in CoO_*x*_ matrices was synthesized. Liang et al. [110] prepared Au-ZnO nanospheres with Au NPs in different particle size encapsulated in ZnO nanospheres through laser irradiation of the Au target and Zn target in liquid, respectively. Otherwise, Liu and Du et al. reported the synthesis of RuAu single-atom alloys (SAAs) [111] and RhO_2_ clusters embedded in the surface of Rh nanoparticles [112] by PLAL technique, the strong quenching effect played an important role in fabricating metastable nanostructures with novel properties (Fig. 8a). The ternary metal alloy AuAgPt was developed by laser-irradiation of chemically synthesized Au@AgPt yolk-shell nanocubes in liquid to induce alloying (Fig. 8b) [113]. In addition, laser driven the plasma resonance absorption performance for noble metal nanoparticles was applied to realize some plasmon-driven chemical process. For instance, Pan et al. [114] reported that through two steps of the first synthesis of Au nanoparticles by PLAL of Au plate and then photochemical reaction between Au nanoparticles and CuCl_2_ under 532 nm pulsed laser, the Cu nanoclusters were achieved due to the reduction of Cu^2+^ ions by plasmon-generated “hot electrons” from Au.

In terms of the synthesis of metals oxides and hydroxides by PLAL, Du et al. synthesized an N-doped graphene oxide by PLAL of a solution composed of ammonia and graphene oxide, which achieved a high pyridinic N dopant (51%) (Fig. 8c). The PLAL process provided a simple and fast approach to prepare N-doped graphene oxide compared with the conventional method, and the obtained N-doped GO exhibited an improved electrochemical HER performance [115]. Bao et al. [116] ablated CoO micropowders by femtosecond (fs) laser pulses in pure water, and the generated CoO nanoparticles suspended in water were collected directly for solar water-splitting. Müller et al. [118] reported the crystalline Co_3_O_4_ nanoparticles (< 5-nm) [117] and [Ni − Fe]-layered double hydroxides (LDHs) as highly active catalysts for oxygen evolution. Co_0.75_Ni_0.25_(OH)_2_ nanosheets with high-density pores via PLAL process were similarly realized (Fig. 8d) [119]. Kim et al. [120] reported thin NiFe LDH nanostructures on 1D-CdS nanorods synthesized by using PLAL. The efficient carrier transport attributed to heterostructures greatly inhibits the recombination of carriers.

Ternary oxide nanocrystals (TONs) have received increasing attention for their inherent optoelectronic properties, such as good electrical conductivity and multiple active sites; thus, they have been well exploited for energy and catalytic applications [121, 122]. However, adoption of surfactants or growth-guiding agents for traditional synthetic methods will decrease the charge transport in products and hence degrade the device performance. Therefore, PLAL provides an attractive, imperative, and green approach to synthesize TONs. Zeng et al. reported a universal PLAL and subsequent hydrothermal growth for various TONs synthesis. A series of TONs, including Zn_2_SnO_4_, NiCo_2_O_4_, Zn_2_GeO_4_, ZnFe_2_O_4_, Fe_2_GeO_4_, and ZnMnO_3_ were synthesized, which confirmed the universality of this method. During the synthesis process, the PLAL-generated highly reactive species, such as metal ions, clusters, H^+^ and OH^−^ will facilitate the formation of various TONs in the subsequent hydrothermal process [123]. Based on this work, Zeng et al. prepared Zn_2_SnO_4_ nanocrystals with an average size of 140 nm by PLAL and subsequent hydrothermal synthesis to construct high-performance nanoscale photodetectors [124]. Yang et al. [125] reported a defective α-Ag_2_WO_4_ nanorods prepared by PLAL. The thermodynamic disequilibrium created by laser ablation combined with the weak bond energy of Ag_2_WO_4_ induced abundant [WO_6_] cluster distortions into the crystal lattice. Similarly, the hydrogen-interstitial CuWO_4_ nanomesh was prepared by PLAL for the application of single-component full spectrum-active photocatalysts for hydrogen evolution [126]. Wang et al. [127] reported the laser synthesis of ligand-free La:BaSnO_3_ nanocrystals by PLAL, and by embedding this laser generated nanocrystals in BiVO_4_ photoanode matrix, an enhanced charge transport for photoelectrode was achieved (Fig. 8e).

In terms of the synthesis of molybdenum sulfide by PLAL, spherical MoS_2_ nanoparticles with onion-like structure and internal shrinkage cavities can be prepared through laser ablation of MoS_2_ target in deionized water [128]. Lee et al. [129] prepared a high concentration of few-layer MoS_2_ via laser exfoliation of bulk MoS_2_ in liquid, the S vacancy and crystal phase transformation were controlled and a dramatically improved electrocatalytic activity toward HER was obtained. Furthermore, the author demonstrated that the presence of protons from solvents with low *pK*_a_ values was conducive to creating S vacancies by extracting S atoms to form H_2_S, and simultaneously facilitated the 2H-to-1 T phase transition of MoS_2_ by sliding S atoms. Amorphous molybdenum sulfide (a-MoS_*x*_) with abundant defect-rich active sites was synthesized by the femtosecond laser ablation of ammonium tetrathiomolybdate aqueous solution with PLICN (Fig. 8f) [130], which was reviewed in the following as the “bottom-up” method. Besides of MoS_2_, very recently, Du et al. [131] used C and O co-doped BN nanospheres as the precursor for PLAL treatment, it demonstrated that the laser-modified boron nitride exhibits unique electrical conductivity and high corrosion resistance under oxidizing conditions, which attributed to the interlayer B–B dipolar interaction.

In contrast to the “top-down” synthesis of PLAL on bulk targets, the “bottom-up” method of PLICN is also a promising liquid-based laser strategy for nanomaterial design [133]. Liang et al. utilized the photo-excited electrons generated by laser processing SnO_x_ in a PtCl_6_^2+^ solution, and hybrid catalysts composed of Pt clusters anchored on the surface of SnO_2_ were obtained, which presented high activity and long-term durability for methanol oxidation [134]. Zheng et al. [102] proposed two distinct reaction pathways of a Co^2+^, Ni^2+^, and Mn^2+^ ion-containing aqueous solution irradiated by lasers with different wavelengths (532 or 1064 nm). The different laser wavelengths resulted in different product formation mechanisms. As demonstrated, the ionization of water molecules to produce reducing species such as solvated electrons (e_aq_^−^) and hydrogen radicals (H·) in the solution dominantly occurred when the 532 nm laser was utilized, and hollow (Ni_0.18_Mn_0.45_Co_0.37_O_*x*_) metal oxide nanoparticles as the final product were formed after the reduction of metal ions by these reducing species and further laser heating. While the 1064 nm laser mainly induced vibration of water molecules, then the stretched OH groups of water molecules facilitated the hydrolysis reaction of metal ions and led to the formation of ([Ni_0.15_Mn_0.15_Co_0.7_(OH)_2_](NO_3_)_0.2_·H_2_O) hydroxide nanostructures. Recently, Liu et al. [135] reported a laser-assisted, continuous, solution route for the simultaneous reduction and modification of graphene oxide with Pt, PtPd alloys, RuO_2_ and MnO_x_ nanoparticles, which demonstrated the versatility of PLICN in synthesizing functional nanoparticle-modified graphene materials.

Laser-based photohydrothermal synthesis of metal oxides was another kind of PLICN technique. Digital ZnO nanowires arrays were achieved by this rapid and one-step selective digital direct growth method, which was realized by localized temperature increase after the thin metal layer absorbed the focused laser, and then induced rapid photohydrothermal reaction of Zn(NO_3_)_2_ solutions near the focal spot region [136, 137]. However, the spatial size was generally limited by the thermal diffusion and the size of the focused laser spot. Ko et al. grew ZnO or TiO_2_ nanowires on a selected area that was even smaller than the laser focus size by laser-based photohydrothermal synthesis. The size of laser-induced temperature field can be effectively reduced through reducing the physical dimension or decreasing thermal conductivity of the laser absorption layer substrate. A smaller nanowire array on PI substrate instead of metal absorption layer can also be successfully synthesized, due to the visible-light absorption capacity and low thermal conductivity of PI [138].

Besides the metal oxides and metal hydroxides, the PLICN technique was also used for other components. For example, Wang et al. [139] prepared Cl-functionalized carbon dots (Cl-CDs) in chlorobenzene via simple laser treatment, which were embedded in perovskite films to regulate both the films morphology and the electronic structures. An improved performance for perovskite solar cells was achieved. Du et al. [122] firstly prepared c-BN NPs with an average size of 3.5 nm at ambient temperature and pressure by exploiting a laser-induced photochemical effect on a dioxane solution of ammonia borane. Beyond that, Irannejad et al. [140] used a focused femtosecond laser to process the air/graphene oxide solution interface to produce rGO gels without any reductive additives, the reduction of the GO and gel formation occurred simultaneously by removing the hydroxyl, carboxyl functional groups and water in the same time. However, when the laser beam was focused inside the graphene oxide solution, only the reduction of GO solution occurred, no gel formed.

##### Laser Synthesis of Nanomaterials by Laser Fragmentation in Liquid

**Fig. 9:**
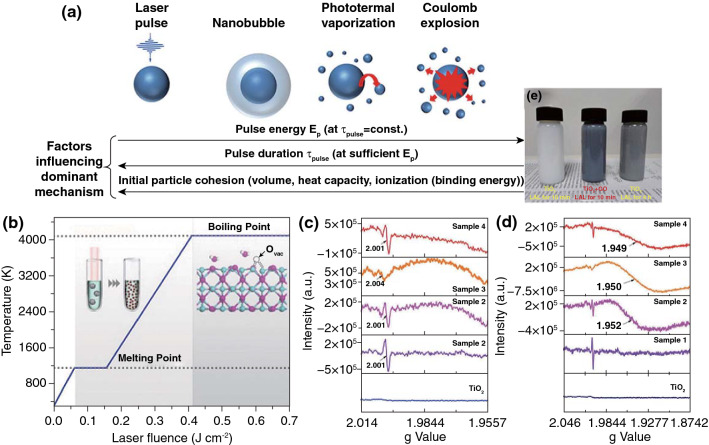
**a** Schematic illustration of the size-reduction and fragmentation mechanisms of plasmonic NPs in a colloidal solution [141]. **b** Preparation of a highly active Co_3_O_4_ catalyst via laser fragmentation [142]. **c, d** EPR spectra of different samples for confirmation of oxygen defects. **e** Photographs of TiO_2_-10 min, TiO_2_ + GO-10 min, TiO_2_-4 h [143]

Laser synthesis of nanomaterials by LFL is induced by the absorption of laser energy with microparticle powder suspensions or nanoparticle colloids. Photothermal vaporization and coulomb explosion are the mechanisms for LFL (Fig. 9a) [141]. To trigger an LFL process in the liquid, commercial solid-state lasers are often combined. Lasers with the pulse width of nanosecond level frequently trigger the photothermal vaporization, while coulomb explosion is triggered by the ultrashort lasers with ps or fs level and is caused by charge repulsion of ionized NPs. LFL can combine both the advantages of size reduction and defect introduction into products, which regulate the electronic structure and lead to a significant improvement of the catalytic activities. Zhou et al. reported that Co_3_O_4_ nanoparticles with a high activity were prepared via the LFL process (Fig. 9b). Ultrafine nanoparticles with abundant oxygen vacancies induced by laser irradiation can significantly improve both electrical conductivity and energy adsorption [142]. Defect-induced electronic reconstruction has also been realized by LFL of photocatalysts and electrocatalysts. G. Yang et al. performed LFL to obtain TiO_2−*x*_-graphene oxide mixtures (Fig. 9c–e). After LFL, the conduction band (CB) moved below 0 eV in the range of − 0.01 to − 0.55 eV, which resulted in a 0.87 eV decrease of the band gap for the visible-light absorption [143]. Zhao et al. employed LFL to liberate the N-doped carbon nanotubes (N-CNTs) confined Co single-atom sites as an efficient selective-hydrogenation material for quinoline. Furthermore, the catalytic conversion of quinoline derivatives with methyl, hydroxyl, and halogen groups into corresponding 1,2,3,4-tetrahydroquinolines was also realized by this liberated catalyst [130]. Musselman et al. [144] utilized the nonresonant intense pulsed laser to fragment and then direct the 2D materials from bulk flakes to assembled nanorod structures. The obtained graphene nanorods, MoS_2_ nanorods, WS_2_ nanorods and BN nanorods suggested the potential in transparent conducting applications.

LAL is summarized to be a “top-down” or “bottom-up” physiochemical method, which usually uses the bulk solid targets (PLAL) or a precursor solution and additional metal absorption layer (PLICN) in liquid condition as the precursors. Therefore, the laser parameters required for LAL are in a wide range, which covering the laser wavelengths from ultraviolet (UV), visible (Vis) to near-infrared (NIR), and the pulse widths from femtosecond, picosecond, nanosecond, microsecond, millisecond, and even extending to the continuous-wave (CW) range. In general, the visible laser with 532 nm wavelength was frequently utilized to process powder precursors in liquid, while the near-infrared laser with 1064 nm wavelength was utilized for target precursor. Ultrafast lasers such as fs or ps laser were usually used for synthesis of nanomaterials through laser fragmentation in liquid, which induced the coulomb explosion process. The effects of the precursors and parameters of laser on the composition nature of obtained nanomaterials in liquid are summarized in Table 3.

**Table 3:** Effects of the precursors and parameters of laser on the composition nature of obtained nanomaterials in liquid

Precursor	Laser source	Parameters	Mechanism/solvent	Products	Applications	References
Ag target	Nd:YAG laser	1064 nm, 7 ns, 15 Hz, 300 mJ	PLAL DI water	Ag nanoparticle	HER	[145]
Au target	Yb:KGW laser	1025 nm, 480 fs, 95 μJ	PLAL DI water	Au nanoparticle	Electrocatalytic glucose oxidation	[104]
AuFe alloys target	Nd–YAG laser	1064 nm, 6 ns, 50 Hz, 10 J cm^−2^	PLAL DI water	Au–Fe nanoalloys	OER	[107]
Au-Pt powder mixture target	KrF excimer laser	248 nm, 17 ns, 20 Hz	PLAL DI water	Au–Pt nanoalloys	4-Nitrophenol reduction	[108]
Co target in K_2_PtCl_4_ solution		60 J cm^−2^	PLAL K_2_PtCl_4_ solution	PtCo nanoalloy embedded in CoO_x_	ORR, OER	[109]
Ru target in HAuCl_4_ solution	Nd:YAG laser	1064 nm, 7 ns, 15 Hz, 30 min	PLAL HAuCl_4_ solution	RuAu single-atom alloy	HER	[111]
Au plate, Zn plate		1064 and 355 nm	PLAL NaOH solution	Au–ZnO nanospheres	Gas sensors	[110]
Au@AgPt yolk-shell nanocubes	Nd:YAG laser	532 nm, 10 ns, 20 Hz, 7.67 mJ cm^−2^	PLAL DI water	AuAgPt alloys	Electrocatalytic methanol oxidation	[113]
Au nanoparticles, CuCl_2_ solution	Nd:YAG laser	532 nm, 6 ns, 10 Hz, 106–107 W cm^−2^	PLAL CuCl_2_ solution	Cu nanoclusters	ORR	[114]
Graphene oxide with ammonia	Nd:YAG laser	1064 nm, 7 ns, 225 mJ	PLAL ammonia solution	N-doped GO	HER	[115]
CoO micropowders	Femtosecond laser	805 nm, 150 fs, 375 mW, 1 Hz	PLAL DI water	CoO nanoparticles	Solar water splitting	[116]
Fe powders, Ni(NO_3_)_2_ solution	Nd:YAG laser	355 nm, 8 ns, 10 Hz	PLAL Ni(NO_3_)_2_ solution	NiFe LDHs	OER	[118]
CoNi alloy target	Nd:YAG laser	1064 nm, 7 ns, 200 mJ, 15 Hz	PLAL KOH solution	Co_0.75_Ni_0.25_(OH)_2_ nanosheets	HER, OER	[119]
Zn plate, Ge plate	Nd:YAG laser	1064 nm, 10 ns, 100 mJ, 10 Hz	PLAL DI water or Zn suspension	Zn_2_GeO_4_	UV photodetectors	[123]
Sn plate, Zn plate	Nd:YAG laser	1064 nm, 10 ns, 100 mJ, 10 Hz	PLAL H_2_O_2_ solution or Sn solution	Zn_2_SnO_4_	UV photodetectors	[124]
Ag_2_WO_4_ powders	Nd:YAG laser	532 nm, 10 ns, 50 Hz, 150 mJ	PLAL DI water	R-Ag_2_WO_4_ nanorods	Visible-light photocatalysis	[125]
CuWO_4_ powders	Nd:YAG laser	532 nm, 10 ns, 10 Hz, 200 mJ	PLAL ethanol	CuWO_4_ nanomesh	Photocatalytic H_2_ evolution	[126]
Bulk MoS_2_ powder	Nd:YAG laser	1064 nm, 7–8 ns, 300 mJ, 10 Hz	PLAL DI water or (CH_3_)_2_CO or (CH_3_)_2_SO or C_5_H_9_NO or C_6_H_14_	Few-layer MoS_2_ nanosheets	HER	[129]
Ammonium tetrathiomolybdate aqueous solution		800 nm, 50 fs, 1 kHz	PLICN aqueous solution	Amorphous MoS_x_	HER	[130]
Ni^2+^, Mn^2+^, Co^2+^ ion^−^containing aqueous solution	Nd:YAG laser	750 mJ at 1064 nm and 350 mJ at 532 nm	PLICN aqueous solution	Ni_0.18_Mn_0.45_Co_0.37_O_x_ (532 nm) [Ni_0.15_Mn_0.15_Co_0.7_(OH)_2_](NO_3_)_0.2_·H_2_O (1064 nm)		[102]
Zn^2+^ aqueous solution	Nd:YAG laser	532 or 1064 nm (depending on the absorption layer metal)	PLICN aqueous solution	ZnO nanowires	UV photodetectors	[136–138]
Chlorobenzene	Nd:YAG laser	355 nm, 8 ns, 10 Hz	PLICN chlorobenzene	Cl-functionalized carbon dots	Perovskite solar cells	[139]
Dioxane solution of ammonia borane	Nd:YAG laser	1064 nm, ns or ms	PLICN Dioxane solution of ammonia borane	c-BN NPs		[122]
EGO solution, metal salt solution (H_2_PtCl_6_, RuCl_3_, Na_2_PdCl_4_, MnCl_2_)	KrF pulsed excimer laser	248 nm, 10 ns, 100, 150, 200, 250, and 300 mJ	PLICN m_isopropanol_:m_acetone_:m_water_ = 2:1:100	Pt, PtPd, RuO_2_ and MnO_x_ nanoparticle-modified graphene	HER OER ORR	[135]
Pristine graphene	Ti:Sapphire laser	800 nm, 35 fs, 0.25 W, 1 kHz	LFL ethanol	Multilayered graphene nanorods	Transparent conducting films	[144]
MoS_2_ or WS_2_ ultrafine powder	Ti:Sapphire laser	800 nm, 35 fs, 0.25 W, 1 kHz	LFL ethanol water (1:1 ratio) solvent	MoS_2_ or WS_2_ nanorods	Transparent conducting films	[144]
BN ultrafine powder	Ti:Sapphire laser	800 nm, 35 fs, 0.25 W, 1 kHz	LFL ethanol water (1:1 ratio) solvent	BN nanorods	Transparent conducting films	[144]

## Laser as Microfabrication Technique for Application

Laser as a microfabrication technique means that the laser is used as an energy source to precisely focus on desirable positions and create patterns locally without affecting the adjacent area [146]. From the major terms, laser synthesis is involved in the laser microfabrication process. Namely, laser as a microfabrication technique is composed of the simultaneous laser synthesis and patterning during the laser processing. Laser microfabrication technique has the advantages of high production efficiency, low cost, stable and reliable processing quality; therefore, it has the good economic and social benefits. In particular, ultrashort pulse femtosecond laser can produce ultrahigh light intensity, it possesses the preponderances of precision and low damage threshold, ultrasmall heating affected area, and can almost precisely process various kinds of materials. In this section, the as-patterned materials after microfabrication through the laser are reviewed, which present high performance in energy storage and conversion devices such as batteries, supercapacitors, sensors and electrocatalytic materials [147].

### Light–Thermal Conversion Devices Fabricated by Laser Technology

Solar-driven water evaporation, wastewater purification and energy conversion are potential green and sustainable technologies; thus, the efficient solar energy harvesting and photothermal conversion play the important role in these applications [148]. In addition, the efficient light-to-heat conversion instead of other forms of energy and heat transferring to the applied process also should be satisfied [149]. It is noteworthy that the light-to-heat conversion efficiency depends greatly on the absorbing material. Therefore, light–thermal conversion materials with high conversion efficiency and broad solar absorption range have attracted widespread interests, such as carbon-based materials, semiconductors, metals and polymers [149–151].

**Fig. 10:**
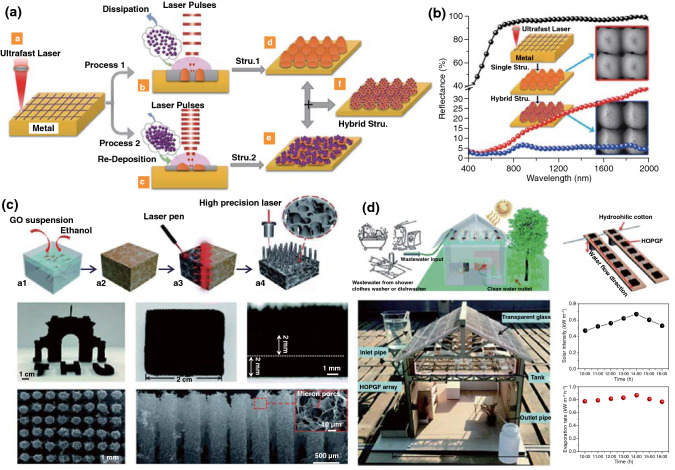
**a** Schematic illustrations of the pulse injection controlled ultrafast laser direct-writing strategy. **b** Hemispherical reflectance of different structures in the UV–Vis–NIR region [156]. **c** Schematic illustration of the preparation of highly vertically ordered pillar array of graphene framework (HOPGF) and the cross-sectional scanning electron microscopy (SEM) images of HOPGF. **d** Schematic illustration of a house supplying clean water based on SSG and the photograph of a laboratory-made house model under the sunlight at Beijing [157]

For the light–thermal conversion materials, not only excellent optical and thermal properties are essential, economic practicality and manufacturing simplicity on a large scale should also be taken into account. The laser processing technique provides a reliable and cost-effective strategy for fabricating nanomaterials with broad-spectrum solar energy absorption on a large scale. In particular, these laser-microfabricated materials are widely applied to various photothermal conversion, anti-reflection and light harvesting applications [152, 153]. By successively constructing microstructures and nanostructures via ultrafast laser patterning and subsequent thermal oxidation, an infrared antireflection nanowire array was obtained on a Cu surface [154]. Attributed to the light trapping by the microstructures, as well as the enhanced photophonon dissipation by the nanowire structure, the ultralow reflectance of 0.6% was achieved for the infrared light with a 17 μm wavelength. Furthermore, Fan et al. also fabricated the cauliflower-shaped copper nanostructures by laser direct writing, which exhibited extremely high broadband omnidirectional absorption of sunlight with high water evaporation efficiency [155]. And then, a universal strategy for fabricating micro/nanohybrid antireflection structures on different metal surfaces by laser direct-writing method was proposed by this group (Fig. 10a, b), the minimum reflectance on W, Cu, and Ti substrates at ultraviolet to near-infrared spectrum reached 2.5%, 1.4%, and 0.29%, respectively [156].

Carbon-based photothermal materials, including graphene, carbon nanotubes, carbon black and graphite, have been used for highly efficient full-spectrum solar absorption materials. Creating a nanostructure with a 3D framework of pores and arrays can reduce light reflection and minimize angular dependence of the incident light. Qu et al. prepared an efficient three-dimensional solar steam generation (SSG) material platform through laser 3D printing technology (Fig. 10c). The as-fabricated graphene framework with a highly vertically ordered pillar array enlarged both the available evaporation area and free space within the graphene framework (Fig. 10c), which resulted in an extremely high evaporation rate of 2.10 kg m^−2^ h^−1^ under 1 sun irradiation (Fig. 10d) [157].

By utilizing the photothermal effects, Zheng et al. [158] proposed an optothermally gated photon nudging (OPN) technique under laser processing. By virtue of an optothermal surfactant thin layer to regulate the interaction between particles and the substrate, the manipulation of colloidal particles into any desired pattern positions with optical scattering force was achieved. However, the orientational control of anisotropic nanoparticles and sub-20 nm position accuracy remained challenging due to the optical diffraction limit.

### Battery and Supercapacitors Fabricated by Laser Technology

Two kinds of typical electrochemical energy storage (EES) device, including batteries and supercapacitors, have been increasingly developed by researchers. However, the application of EES devices has often been limited by poor mechanical performance, low power density, high cost, and short cycle life of devices [159]. The laser microfabrication technologies provide efficient direct-writing processed and novel, low-cost, reliable, environment-friendly and template-free patterning methods to design and fabricate high-performance electrodes for high-quality energy storage devices.

#### Supercapacitors

**Fig. 11:**
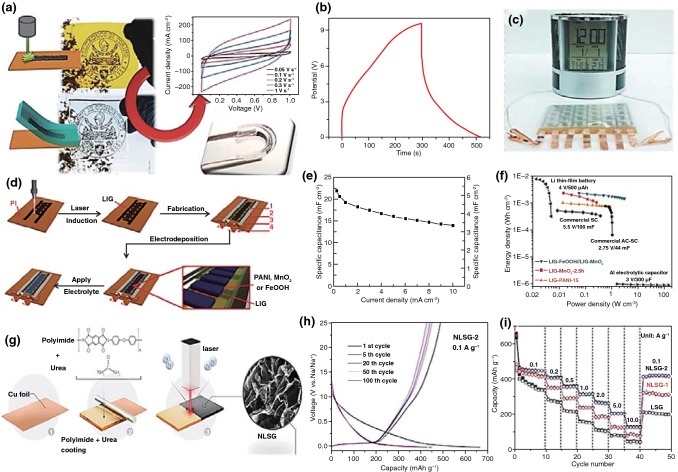
**a** Manufacturing and processing of laser-induced graphene electrodes for highly stretchable supercapacitors [167]. **b** Galvanostatic charge/discharge curves of the MSC array at the current density of 0.5 mA cm^−2^. **c** Electronic pen container with music alarm was powered by the 6S × 5P AMSC array [169]. **d** Scheme of the fabrication of MSCs with LIG–MnO_2_, LIG–FeOOH, or LIG–PANI. **e** Areal and volumetric specific capacitance of LIG–FeOOH//LIG–MnO_2_ over a current density range of 0.25–10 m cm^−2^. **f** Ragone plots of LIG–MnO_2_-2.5 h, LIG–PANI-15, and LIG–FeOOH//LIG–MnO_2_, compared with commercially available energy storage devices [174]. **g** Schematic to illustrate the process flow used to fabricate the nitrogen-doped 3D graphene directly onto Cu foil through laser scribing. **h** Galvanostatic charge/discharge profiles of NLSG-2 electrode at 0.1 A g^−1^. **i** Rate performance of LSG, NLSG-1, and NLSG-2 electrodes at different current densities [54]

Supercapacitors (SCs), also known as electrochemical capacitors, were considered as one of the most promising EES systems due to their fast charge–discharge capacity, long cycle life, high power density and safety [160–162]. In recent years, laser-based technologies have been used for SCs in laser-derived graphene microfabrication and preparation from graphene oxides [163, 164] or polymers [32, 165, 166]. In 2012, using LightScribe DVD burner, Kaner et al. [25] directly laser-reduced the GO to laser-scribed graphene (LSG) in one step for scalable fabrication of SCs, which led to the microfabrication of many LSG electrodes and flexible SCs. For example, they also used a LightScribe DVD burner to interdigitate graphene electrodes on the disc, and more than 100 micro-supercapacitors can be produced on a single disc in 30 min or less [26]. Noteworthily, the produced devices are extremely thin and are completely flexible. Lamberti et al. [167] reported a type of porous laser-induced graphene with a large surface area and good electrical conductivity, which was fabricated by a CO_2_ laser direct-writing PI sheet. As illustrated in Fig. 11a, after transferring the obtained LIG onto PDMS as the stretchable and flexible electrodes to fabricate LIG/PDMS supercapacitors, stable mechanical and electrochemical properties under highly stretching conditions were achieved. Moreover, laser microfabrication technologies also have been utilized to fabricate micro-supercapacitors (MSCs) with outstanding capacitive performance, which allowed massive construction of microelectrode patterns on a flexible substrate [168, 169]. With this technique, the five parallel rows of six series-connected MSCs was simultaneously fabricated and displayed the high voltage output of 9.6 V, successfully powering the electronic pen container with music alarm [169] (Fig. 11b, c). It also demonstrated that the compact hybrid 3D MSCs array integrated with solar cells contributed to efficient solar energy harvesting and storage. Besides, carbon–metal oxide nanocomposites, such as MnO_2_/graphene [170], graphene/RuO_2_ [171], TiO_2_/graphene [172], LIG/NiO/Co_3_O_4_ [173], and metal/oxide [170], have been prepared via laser or laser combining with other fabrication processes, which can efficiently improve power density and energy density of SCs. Li et al. [174] demonstrated that the laser microfabrication technique was also widely applied in pseudocapacitive materials. As shown in Fig. 11d, hybrid composites of pseudocapacitive materials, such as MnO_2_, FeOOH and polyaniline (PANI) integrated with LIG, were achieved by combining the laser microfabrication for patterns with the subsequent electrodeposition process. At the fixed current density of 0.25 mA cm^−2^, the volumetric and areal specific capacitance of the LIG-FeOOH//LIG-MnO_2_ asymmetric device was calculated to be 5.4 F cm^−3^ and 21.9 m F cm^−2^, respectively (Fig. 11e). Through comparison, the presence of pseudocapacitive materials in MSCs benefits for a greater electrochemical performance than some commercial SCs, and even comparable to Li thin-film batteries (Fig. 11f), which was ascribed to the high capacitance of metal oxides and the electrical conductivity of LIG. In resent report, Wu et al. firstly developed conductive MOF [175] grown on LSG selectively as electrodes for electrochemical micro-supercapacitors with greatly enhanced performance. Teng et al. [176] combined electrophoresis deposition with laser microfabrication to pattern the activated mesophase pitch carbon integrated with a gel electrolyte on the substrate, which was utilized for the on-chip assembly of micro-supercapacitors. In addition, Wang and Fang et al. [177] fabricated a femtosecond laser-etched MXene MSCs with double-side configuration via arbitrary on- and through-substrate connections of MXene MSC units. The MXene double-side MSC fabricated by the series connection of 12 spiral pattern MXene MSC units with interdigital electrodes of 10 μm width interspace can output a large working voltage of 7.2 V. In contrast to the complexities of necessitating masks and the wet-etching process for photolithography, laser microfabrication process is low-cost, quick, and readily scalable.

#### Battery

As well known, batteries with fast surface redox reaction always have high energy density but low power density and poor cycling performance. Such limitations restrict their applications as independent energy storage devices. Recently, the laser microfabrication technology, which attracted special attention, has been deemed promising to minimize the fabrication costs, increase the operational lifetime and enhance the power density performance of batteries [178]. Laser-assisted chemical vapor pyrolysis (LaCVP) [179] was used to synthesize the active materials in batteries. For instance, Muna et al. [180] reported preparation of the Si-based nanostructured electrodes using LaCVP as an anode material in advanced lithium-ion batteries for potential practical usages, which achieved high performance, dramatic cyclability and fast scalable production in high purity. Zhang et al. [181] used the laser-induced MnO/Mn_3_O_4_/N-doped-graphene hybrid as binder-free anodes for lithium-ion batteries, which performed a high reversible capacity of 992 mAh g^−1^ at 0.2 A g^−1^ and excellent rate capacity of 365 mAh g^−1^ at 2.0 A g^−1^, as well as a high cycling stability. Laser fabrication technologies have also been used for electrochemical storage of Na-ions batteries. Zhang et al. [182] demonstrated that CO_2_ laser irradiation was a fast and effective way to fabricate hard carbons for application in Na-ion batteries, where the structural organization degree was increased by laser microfabrication through producing locally ordered turbostratic carbon. Furthermore, Zhang et al. [54] fabricated N-doped 3D graphene directly bonded to the current collectors as anode for sodium-ion batteries, which was manufactured through a direct laser-scribing process under nitrogen gas atmosphere (Fig. 11g). Because of the high concentration of nitrogen doping and high electrochemical surface area of LSG, the laser-fabricated electrode exhibited excellent electrochemical performance with an initial discharge capacity of 659 mAh g^−1^ and a recovered charge capacity of 485 mAh g^−1^ (Fig. 11h). In addition, Na-ion capacities up to 425 mAh g^−1^ at 0.1 A g^−1^ and 148 mAh g^−1^ at 10 A g^−1^ were achieved with excellent rate capabilities and cycling durability (Fig. 11i). This work points out a promising route for fabrication of metal-ion capacitors directly on current collectors via optimized laser conditions. It was noteworthy that, only a few works on the application of laser processing in batteries were reported. Hence, laser processing in batteries, especially for the fabrication of active materials directly on current collectors via the optimized laser microfabrication technique should be extensively explored for electrochemical storage of different metal cations in the future.

### Sensor Devices Fabricated by Laser Technology

Because of the instantaneous local high temperature and pressure environment occurred on the surface of materials when irradiation by laser technology, the surface structures of laser-treated materials are porous and fluffy, which have some unique advantage to be the sensitive layer of pressure or gas sensor. Furthermore, the pattern array device is easy to be obtained by laser microfabrication method. Therefore, most devices fabricated by laser technology are suitable to be used as sensor devices [183]. In this section, different sensor devices fabricated by laser technology were reviewed.

#### Force Sensor

**Fig. 12:**
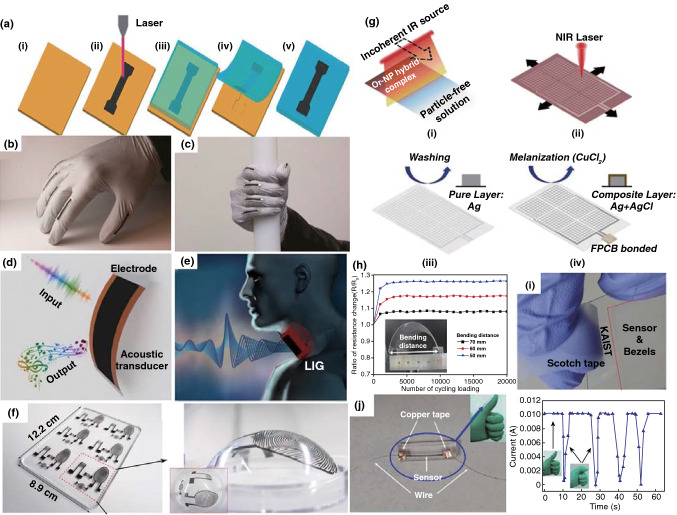
**a** Schematic of the fabrication process for stretchable carbon nanocomposite using laser pyrolization of polyimide; **b, c** Human finger motion detection with stretchable carbon traces [185]. **d** LIG has the ability of emitting and detecting sound in one device; **e** Artificial throat can detect the movement of throat and generate controllable sound, respectively [186]. **f** Interdigitated transducer geometries produced by laser ablation of cast carbon–silicone bonded to an elastomer [188]. **g** Overall fabrication steps of the touch device using the laser process. **h** Cycling bending test for the laser-processed touch sensor, as a function of bending distance. The bending rate was 500 mm min^−1^. The inset is a photograph of bending test setup. **i** Peeling test using a conventional scotch tape after 100 times [191]. **j** Photograph and human motion detection of finger with flexible sensor based on copper electrode [192]

Force sensors are widely used in various fields, such as smart textiles, wearable electronics, artificial intelligence robots and structural health monitoring [184]. As mentioned in Sect. 2.1, carbon materials were commonly fabricated by laser ablation. Generally, they were also nanomaterials for application in force sensors. Rahimi et al. reported a unidirectional strain sensor based on transfer and embedment of carbonized patterns fabricated by laser microfabrication of thermoset polymers (Fig. 12a), which obtained the stretchability of up to 100%, and sensitivity of up to 20,000. They used this technique for realizing measurement of finger motion in real time (Fig. 12b, c) [185]. Tao et al. fabricated an intelligent artificial throat using a similar method. The graphene foam fabricated through laser pyrolysis was the sensitive layer for AI throat, which could not only generate sound by thermal acoustic but also detect sound by piezoresistive effect (Fig. 12d, e) [186]. Laser-induced carbon materials with 3D pattern structures by two-step fabrication extended their applications in force sensors [187]. For example, Tour et al. utilized the 3D patterned graphene foams by laser laminated object manufacturing in strain sensors to monitor the pulse fluctuation frequency [37]. Araromi et al. patterned PDMS-carbon composite layers by laser ablation, the high-resolution and mechanically robust electrode was achieved for stretchable silicone elastomer actuators and sensors. They validated that the devices fabricated by laser microfabrication can realize the large area of up to A4-size and high sensitivity (Fig. 12f) [188]. Luo et al. [27] used the scribing process for the reduction and patterning of graphene oxide film to realize LSG sensor fabrication. The versatility of LSG sensors provided a promising strategy for monitoring and mapping large-scale deformation and strain distribution of polymeric composites. Nag et al. used the laser-patterned composite of multi-walled carbon nanotube (MWCNT) as conductive film and PDMS as substrate for the flexible and wearable sensor application, which realized monitoring of respiration and limb movements [189]. Besides of the composite of MWCNT and PDMS, Nag et al. also tried to use Al film as the conductive layer on PI substrate. The laser microfabrication-induced aluminum interdigitated electrodes for tactile sensors were sensitive to finger pressure [190].

Except for the carbon materials, the patterned non-carbon electrodes by laser microfabrication also have excellent sensitivity for sensors. Son et al. performed organometallic metallization using laser thermochemical treatment (Fig. 12g). Attributed to the advantage of device patterning by laser, a high pressure resolution can be obtained by sensor arrays (Fig. 12h, i) and this laser patterning method was crucial for high-yield industrial production [191]. Bai et al. prepared the patterned devices based on copper electrode by laser microfabrication, high sensitivity and mechanical robustness were realized when it was applied as a touch switch (Fig. 12j) [192]. Arnaud Spangenberg et al. [193] used the near-infrared fs laser irradiation to prepare crystallized TiO_2_ and TiO_2_/C nanocomposite microstructures as microsized pressure sensors. When using the AFM tip to gradually approach the sensor, the pressure sensing exhibited the sensitivity at the microscale from a single line and a repeatable response.

#### Gas Sensor

Gas sensors, such as CO detectors, humidity sensors or other harmful gas sensors, play an important role in health monitoring. Therefore, gas sensors are significant for our daily lives. Laser microfabrication technology also derived many excellent researches in this field [194].

**Fig. 13:**
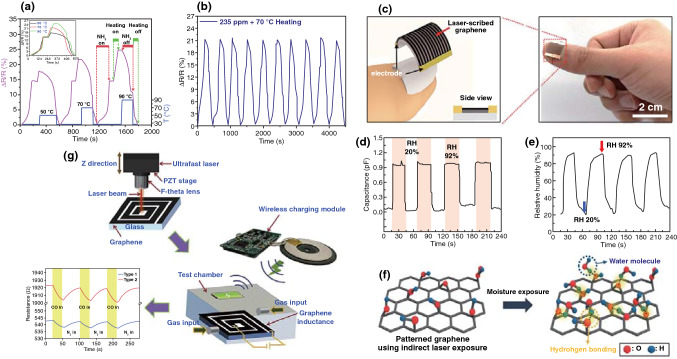
**a** Normalized real-time resistance response/recovery behavior of the sensor to 235 ppm NH_3_ at various desorption temperature from 50 to 90 °C. **b** Real-time cycling response of the sensor to 235 ppm NH_3_ gas at 70 °C [195]. **c** Schematic view and digital image of the laser-scribed rGO-based flexible humidity sensor attached on a nail. **d** Real-time signal responses as measured cyclic capacitance changes from the laser direct-writing (LDW) GO-based humidity sensor, exposed to RH in the range of 20–92% at 30 s intervals. **e** Monitored relative humidity (RH) changes collected from the real-time data logger correspond to the graph in **d**. **f** Schematic drawing of absorption process of water molecules by hydrogen bonding on the partially reduced GO surface after moisture exposure [196]. **g** Schematic of the gas detection setup [198]

As mentioned above, the laser-induced structure of carbon nanomaterials is porous and floppy, which means that the devices based on laser-induced carbon structure are also suitable for gas sensors [34]. For instance, Wu et al. fabricated a graphene-based NH_3_ sensor by one-step laser treatment of PI. The response/recovery time of this NH_3_ sensor were 214 and 222 s for detection of 75 ppm NH_3_, respectively. In addition, the sensitivity and cyclic stability were greatly improved under the heating temperature of 70 °C (Fig. 13a, b) [195]. The laser-fabricated molybdenum carbide/graphene composites mentioned above can not only apply for paper-based supercapacitors, but also for the gas sensors and electrochemical ion detectors applications [81]. Park et al. used the interdigitated rGO obtained by one-step laser treatment as a humidity sensor pasted on fingernail (Fig. 13c–f), where wearable monitoring was realized [196]. Drmosha et al. reported a Pt-loaded rGO/ZnO gas sensor fabricated by laser, the significant selectivity and response of about 99% toward hydrogen with a low concentration of 400 ppm was achieved, which was 5 and 10 times higher than those of the rGO/ZnO composite and pure ZnO, respectively [197]. In addition, Chang et al. precisely fabricated the spiral graphene pattern electrodes as the sensing test region by double scanning of the picosecond laser. The obtained gas sensor had high detection sensitivity to the CO, H_2_O, and air (Fig. 13g) [198].

#### Other Devices Fabricated by Laser Technology

**Fig. 14:**
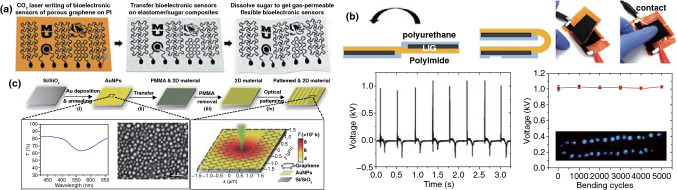
**a** Schematic illustration of the fabrication process for bioelectronic sensing systems [199]. **b** Performance of a flexible metal-free TENG [200]. **c** Schematic flowchart of OTNL process for 2D materials patterning [206]

One-step laser carbonization provided more possibilities for other flexible devices. For example, Yan et al. reported a kind of electrophysiological sensor composed of laser-patterned porous graphene as the sensing components and sugar-templated silicone elastomer sponges as the substrates (Fig. 14a). The sensors exhibited much higher water vapor permeability (18 mg cm^−2^ h^−1^) than those without pores, which solved the problem of limited gas permeability. More importantly, this strategy used simple and effective laser processing instead of thin-film deposition, photolithography, or other complex procedures [199]. Stanford et al. fabricated a high output power triboelectric nanogenerator (TENG) by CO_2_ laser treated polymers (Fig. 14b) [200]. High output power TENG through laser-treated surface of friction materials was also fabricated by Huang [201].

Most of the sensor devices transformed the strain/pressure or chemical energy to the electric energy [202]. However, the stretchable organic light-emitting device (OLED) was another sensor device that was mechanically insensitive to fatigue strain. OLED has become a promising alternative in electronic skin and deformable displays. Sun et al. developed a stretchable OLED with an ordered drape pattern through laser-programmable microfabrication, the stretch-release circulation can be controlled and the maximum efficiency of 72.5, 68.5, and 70 cd A^−1^ at mechanical strain of 0%, 40%, and 70%, respectively, was achieved [203].

Besides the applications of energy storage and sensors, nanostructures patterned by laser microfabrication technology for biological applications are receiving increasing attention, especially for the study of adhesive interactions between cells and nanostructured interfaces. Healy et al. fabricated nanocraters in quartz through the pulsed femtosecond laser; then, they studied the effect of spatial distribution and features of the nanocraters on cell migration, morphology, and spatial organization. It demonstrated that this laser surface-patterning approach provided a simple proposal for controlling cell behavior [204].

In addition, Jo et al. reported the writing of integrated circuits on a 2D semiconductor using a visible laser. In particular, by scanning the laser over metal patterns deposited on 2H-MoTe_2_ semiconductor layers, the spatially resolved doping with controlled dopant profiles and concentrations was achieved. This allowed us to directly write various circuitry in designed patterns, including arrays of n–p–n (p–n–p) bipolar junction transistors (BJTs) and p–n diode discs [205]. Zheng et al. [206] developed a new optothermoplasmonic nanolithography (OTNL) technique. By virtue of the plasmon-enhanced light absorption and localized photothermal effect in deposited Au nanoparticles, the on-demand, low-power and desired patterning of 2D materials (graphene and MoS_2_ monolayers) was achieved through a continuous-wave laser irradiation (Fig. 14c). It demonstrated that thermal oxidation for graphene patterns and sublimation for MoS_2_ patterns under the laser-induced temperature field can lead to direct etching of the corresponding atomic layers.

### Electrocatalytic Materials and Electrodes Fabricated by Laser Technology

High efficiency of the electrolyzer system is dependent on the electrocatalytic materials with high activity. It is noted that the surface structure and the surface area play an important role in improving the activity of electrocatalytic electrode materials for hydrogen evolution reaction (HER), oxygen evolution reaction (OER) or oxygen reduction reaction (ORR). Various techniques have been used to construct the surface structure of electrocatalytic electrodes [207–209], especially by using the template synthesis [209]. However, the removal of the template was tedious, and the laser microfabrication technique provided an alternative method to construct different surface microstructures of electrocatalysts. With regards to the relationship between the high HER performance and laser microfabrication technology, it can be attributed to two factors. On the one hand, powder-like electrocatalysts prepared by laser ablation in liquid usually possess the high electrocatalytic activity due to the laser-induced defects and the large specific surface area for exposing more active sites on the electrocatalysts. On the other hand, for the 3D electrocatalytic electrodes fabricated by the laser microfabrication technique, the high performance can be attributed to the different surface microstructures constructed by laser. During the laser microfabrication process, the hydrophilic surface, the large specific surface area for exposing more active sites and even the formation of a more active nanomaterials can be achieved, which are all preferable to the improvement of the electrocatalytic performance.

#### Electrocatalytic Hydrogen Evolution Reaction (HER)

**Fig. 15:**
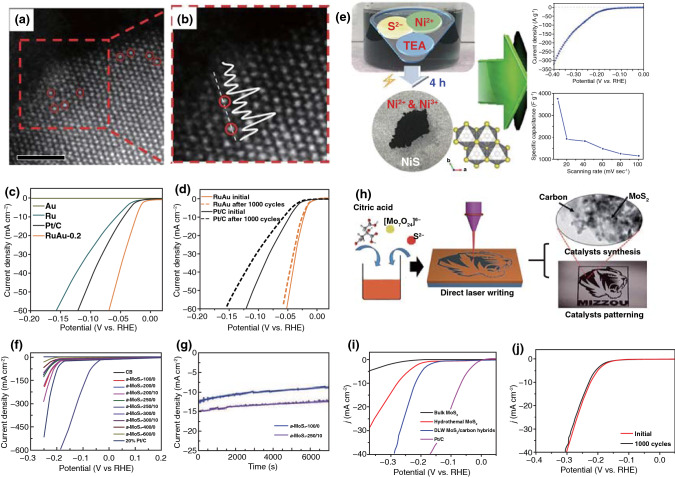
**a** Atomic-resolution HAADF-STEM image, Au atoms (marked by red circles) are uniformly distributed throughout the particle. Scale bar: 1 nm. **b** Magnified image in red dotted rectangle of **a**, Au atoms were marked with red circles. The white solid curve is the integrated pixel intensity along the white dotted line. HER activity and stability in 1 M KOH solution. **c** Linear sweep voltammetry (LSV) polarization curves (iR compensated) at scan rate of 5 mV s^−1^; **d** durability test [111]. **e** Nickel sulfide nanostructures prepared by laser irradiation for efficient electrocatalytic hydrogen evolution reaction and supercapacitors [214]. **f** Polarization curves of CB, the a-MoS_x_ materials, and 20% Pt/C catalysts in 0.5 m H_2_SO_4_. **g** Results of the durability tests for the a-MoS_x_-100/0 and a-MoS_x_-250/10 materials [132]. **h** Scheme of the LDW method in fabricating arbitrary patterns composed of MoS_2_/carbon hybrids. **i** Polarization curves of Pt/C, bulk MoS_2_, hydrothermal MoS_2_, and laser-induced MoS_2_/carbon hybrids. **j** Durability of laser-induced MoS_2_/carbon hybrids [217]

Electrocatalysts with high activity and durability for HER are crucial for the practical demands of electrochemical water splitting. The laser synthesis and microfabrication techniques provide a new avenue toward fabricating nanostructures with novel properties due to the strong quenching effect [210]. As mentioned in Sect. 2.2.2, Du et al. reported a novel active Ag electrocatalyst [145] and RuAu single-atom alloy electrocatalyst [111], which was fabricated by laser ablation in liquid. The HER electrocatalytic activities outperformed Pt/C. As reported, the Ag nanoparticle electrocatalyst exhibited an extremely high activity for HER (a low overpotential of 32 mV at 10 mA cm^−2^ and Tafel slope value of 31 mV dec^−1^) in acidic electrolyte, and the transformation from inactive to highly active Ag electrocatalysts was attributed to the improved H absorption energy regulated by laser-induced stacking faults. The prepared RuAu single-atom alloys also exhibited high stability and low overpotential of 24 mV at the current density of 10 mA cm^−2^ in alkaline media (Fig. 15a–d). The high HER performance was attributed to the following factors: Firstly, the strong hydrogen atoms adsorption by Ru matrix can be counteracted by Au with weak hydrogen adsorption. Secondly, the electronic structure of RuAu single-atom alloys can be regulated by the immiscibility between Ru and Au. Lastly, chemically inert Au favors long-term stability during the reaction.

Non-noble metal electrodes with nanostructures microfabricated by laser were also the candidates for effective electrocatalytic electrodes. Röntzsch et al. reported the surface construction of Ni electrodes through the fs pulse laser for highly active hydrogen evolution, which was realized by enlarging the surface area of Ni [211, 212]. An enhancement of HER activity was achieved and the author confirmed that contributions of the microstructure of the redeposited ablation products and the chemical composition could be neglected for the HER activity, only the morphological surface changes with enlarged surface areas contributed to the HER activity. Also utilizing the ultrashort-pulse laser, the same group reported the laser process-induced micro- and nanostructured Ti surfaces loading Pt by sputter deposition as HER electrode for alkaline water electrolysis [213]. It was demonstrated that the dramatically improved HER performance was ascribed to the significant increase in specific surface area as well as the rapid gas bubble detachment resulted from superhydrophilic and superwetting properties. In addition, Zheng et al. [214] reported a nickel sulfide nanostructures prepared by nanosecond pulsed laser ablation of an aqueous precursor solution under ambient condition. The superb electrochemical performances for electrocatalytic HER and supercapacitors were achieved (Fig. 15e), which exhibited a low overpotential (− 159 mV vs. RHE at 10 A g^−1^), lower Tafel slope (218 mV dec^−1^) and long-term durability. Another transition metal sulfide, MoS_2_ was regarded as one of the most effective substitutes to the noble metal Pt for HER. Thus, the regulation of MoS_2_ with amorphous or crystalline structures and hybrid structures by laser attracted much attention. Tran et al. [215] investigated the crystallization (c-MoS_2_) of amorphous molybdenum sulfide (a-MoS_*x*_) induced by laser, which confirmed that a-MoS_*x*_ had a higher catalytic performance for H_2_ evolution. This result displayed that defect-rich active sites were beneficial to high activity of the HER catalytic performance. The nanosized amorphous molybdenum sulfide (a-MoS_*x*_) with numerous defects and disordered structures by femtosecond laser ablation of ammonium tetrathiomolybdate aqueous solution has been mentioned in Sect. 2.2.2 [132]. It was noteworthy that, large current density of 516 mA cm^−2^ at an overpotential of 250 mV could be achieved by using the optimized a-MoS_*x*_ catalysts (*x* = 2.73) (Fig. 15f, g). In addition, Wu et al. [216] also prepared MoS_2_ quantum dots with defective structure by ultrafast laser ablation. The enhanced HER electrocatalytic performance was achieved on account of the high conductivity, abundant exposed active sites and good hydrophilicity resulted from the laser ablation.

The electrocatalytic performance of MoS_2_ has also been limited by the poor conductivity. Therefore, the hybrid structure of MoS_2_ with other conductive materials has been paid attention. Lin et al. [217] presented a laser direct-writing method to synthesize and pattern MoS_2_/carbon hybrid materials as HER electrocatalysts under ambient conditions, where small-sized MoS_2_ NPs anchored on carbon matrix were in situ synthesized and arbitrary patterns of this hybrid structure on substrates could be designed through computer-controlled laser beams. Compared with MoS_2_ synthesized by conventional hydrothermal method, the laser direct-writing method can not only realize the synthesis of MoS_2_ in a short period of time, but also perform superior HER activity for laser-induced MoS_2_/carbon hybrid materials (Fig. 15h-j). Li et al. [218] prepared Pt-MoS_2_ and Ag-MoS_2_ composites, which exhibited an enhanced activity for HER. The decorated Pt metal nanoparticles contributed to the high HER activity and was reduced by photogenerated electrons of MoS_2_ nanosheets induced by the femtosecond laser.

#### Electrocatalytic Oxygen Evolution Reaction (OER)

The most common OER electrocatalysts are regarded as transition metal oxides and hydroxides. By using laser as the power source under ambient atmosphere, laser ablation synthesis of OER electrocatalysts is facile and feasible. Most of the synthesis methods for these OER electrocatalysts were laser ablation in liquid, as mentioned in Sect. 2.2.2, all of them exhibited excellent OER activity for water oxidation [219, 220]. For example, Co_3_O_4_ nanoparticles with an overpotential of 314 mV at 0.5 mA cm^−2^ [117], CoO–Co_2_O_3_–Co(OH)_2_ multiphase nanoparticles with an overpotential of 100 mV lower than that of the CoO submicron precursor particles [221], NiFe layered double hydroxides with a lowest overpotential of 260 mV at 10 mA cm^−2^ [118], CoO nanoclusters/CoFe LDHs hybrid with an overpotential of 254 mV at 10 mA cm^−2^ [222] and oxygen vacancy-modified CoOOH requiring an overpotential of 330 mV to reach 10 mA cm^−2^ [223]. The performance of most of the OER electrocatalysts synthesized by LAL even outperforms RuO_2_.

**Fig. 16:**
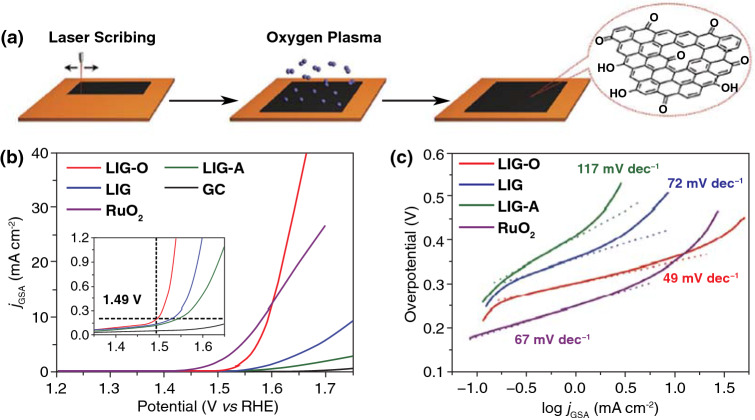
**a** Preparation of LIG-O. **b** LSV curves of LIG-O, LIG, annealed LIG (LIG-A), and a glassy carbon (GC) electrode recorded in 1 m KOH at a scan rate of 2 mV s^−1^. **c** Tafel plots calculated from panel **b** [224]

Besides the transition metal oxides and hydroxides, Tour et al. [224] used laser microfabrication to prepare oxidized laser-induced graphene (LIG-O) as an efficient metal-free OER electrocatalyst (Fig. 16a). The laser-induced graphene was highly porous to expose more active sites for OER. Furthermore, the oxidation of LIG by O_2_ plasma to form LIG-O boosted the activity for OER, exhibiting a low onset potential of 260 mV with a low Tafel slope of 49 mV dec^−1^ (Fig. 16b, c). The same group also prepared Co_3_O_4_ in graphene [80] and NiFe/LIG catalysts [225] for oxygen electrocatalysis, just by depositing the different metal precursors on a preformed LIG surface followed by laser scribing. On account of the addition of transition metal precursors, the OER activity significantly improved.

#### Electrocatalytic Overall Water Splitting

**Fig. 17:**
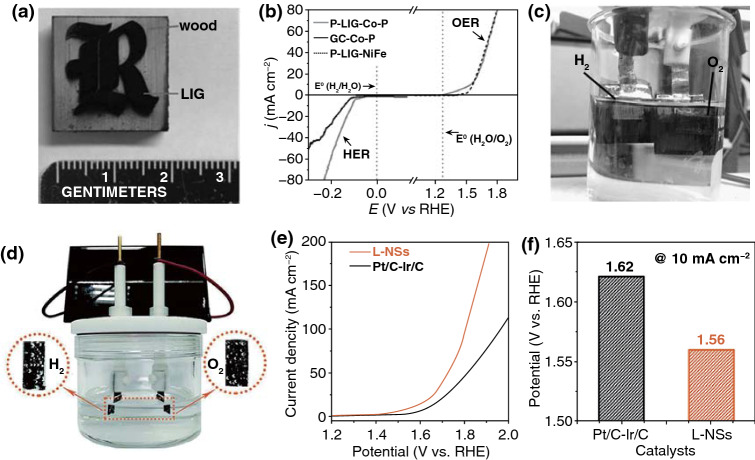
**a** A photograph of LIG patterned into a letter R on pine wood. **b** HER and OER windows (iR compensated) of P-LIG-70 deposited with Co-P or NiFe in 1 M KOH aqueous solution. **c** A photograph shows hydrogen and oxygen bubbling over the P-LIG-Co-P (left electrode) and P-LIG-NiFe (right electrode) surfaces powered by two 1.5 V batteries in series [227]. **d** Optical image of overall water splitting driven by a 1.51 V solar cell. **e** LSV curves of Co_0.75_Ni_0.25_(OH)_2_ nanosheets and commercial Pt/C-Ir/C couple in 6 m KOH for overall water splitting. **f** Potential values at 10 mA cm^−2^ [119]

As depicted above, Tour et al. [226] has made great efforts on oxygen evolution reaction electrocatalysts based on LIG, this group also paid a lot of attention on laser-induced graphene process to fabricate active catalytic electrodes for overall water splitting to generate both O_2_ and H_2_. Firstly, they fabricated an overall water splitting device with HER and OER electrocatalytic electrodes on opposing faces of a commercial polyimide (PI) sheet. The HER active species were formed in situ in the LIG forming process by impregnating the PI film in a Pt-ion solution, and the OER active species were formed by subsequent electrodeposition of CoP/Co_3_(PO_4_)_2_ or Ni_*x*_Fe_*y*_(OH)_2*x*+3*y*_ onto the LIG. As demonstrated, the LIG electrolyzer composed of LIG-Co-P as cathode and LIG-NiFe as anode was employed in 1 M KOH, delivering 1.66 V at the current density of 10 mA cm^−2^ with efficient HER and OER activity. In addition, this group used CO_2_ laser scribing to transform wood into hierarchical porous graphene [227] (Fig. 17a). By varying the electrodeposition materials on the LIG with Co-P or NiFe LDHs, the laser-induced graphene patterned on the wood surfaces could be readily fabricated into HER and OER electrodes for overall water splitting with high reaction activity at low overpotentials (Fig. 17b, c).

Besides the LIG-based electrocatalytic electrode, laser-induced hierarchical oxide nanostructures were also candidates for electrocatalytic overall water splitting. Zhong and Wu et al. [228] manufactured a series of metal oxides (MO_*x*_, M = Ti, Mn, Fe, Co, Ni, Cu, Mo, Ag, Sn, W, and NiFe) with a hierarchical nanostructure on corresponding metal substrates through laser ablation. In particular, the NiO/Ni plate electrocatalysts exhibited the HER overpotential of 121 mV at 10 mA cm^−2^, much lower than that of 279 mV for Ni before laser ablation. Meanwhile, a decreased OER overpotential of 294 mV at 10 mA cm^−2^ than 403 mV for Ni suggested the dramatically enhanced OER activity after laser ablation. Yang et al. [229] developed a surface laser modification approach for fabricating NiCo_2_O_4-d_ with higher Ni^3+^/Ni^2+^ ratio and abundant oxygen vacancies (Vo··) from NiCo_2_O_4_. The laser-modificated NiCo_2_O_4-d_ exhibited a higher electrocatalytic activity for overall water splitting compared with the initial NiCo_2_O_4_. Du et al. [119] used laser-synthesized porous Co_0.75_Ni_0.25_(OH)_2_ nanosheets to catalyze the overall water splitting (Fig. 17d–f), which exhibited a high performance for HER (95 mV@10 mA cm^−2^) and OER (235 mV@10 mA cm^−2^). When using it as both cathode and anode catalysts, an external voltage of only 1.56 V was needed to reach a current density of 10 mA cm^−2^, much lower than that of commercial Ir/C-Pt/C couple (1.62 V). These works demonstrated that using laser ablation to create novel nanostructures with highly active sites for overall water splitting has great application prospects.

#### Electrocatalytic Oxygen Reduction Reaction (ORR)

As mentioned in Sect. 2.1.2, Tour’s group [79] scribed a CO_2_ laser on a metal-complex containing polyimide film and different metal oxide nanoparticles, Co_3_O_4_, MoO_2_, and Fe_3_O_4_ embedded in porous graphene were formed, which were highly active in electrocatalytic ORR. However, it was noted that when the as-prepared metal oxide nanoparticles embedded into porous graphene was applied in ORR, annealing process at 750 °C for 30 min was performed in order to improve the degree of crystallization while maintaining the same crystal phases of metal oxides.

### Other Applications of Materials Fabricated by Laser Technology

#### Corrosion Protection Application

Corrosion of metals has been studied for its disadvantages of causing tremendous disasters and economic loss. Because of the unique high conductivity, impermeability and chemical inertness properties, the graphene coating has become a promising choice for anticorrosion application. However, graphene is easily fabricated on copper or nickel substrates while being difficult to grow on carbon steels. Therefore, Zhong et al. grew graphene by laser microfabrication on the solid carbon coating nickel surface [48] and on normal carbon steel by introducing Ni element into the carbon steel surface [230], which exhibited excellent anticorrosion property. This strategy contained the advantages of fast growth, large-area growth and arbitrary pattern design for graphene synthesis and manufacturing, overcoming the limitation of the CVD method. However, it was noted that both of the wide laser beam size and high laser power density were essential during the graphene synthesis process.

#### Hydrophilic and Hydrophobic Applications

The exciting functionalities of natural superhydrophobic and superhydrophilic surfaces inspired a variety of biomimetic designs. Particularly, the micropatterns combined with wetting states resulted in invigorating applications. By using a fs-laser microstructuring technology, Kostal et al. [231] mimicked the Namib Desert beetle’s elytra, the double hierarchical surface structures with superhydrophilic character (water contact angle < 10°) on glass were firstly fabricated through laser. After a Teflon coating to change the wetting state from superhydrophilic to superhydrophobic (water contact angle > 150°) was applied, selective laser ablation was then utilized to locally expose the superhydrophilic pattern. The fabricated micropatterns exhibited a significantly enhanced fog collection efficiency of 60% higher than blank glass, it was demonstrated that efficient drop accumulation and fast drop removal contributed to this enhancement. In addition, Chen et al. presented the fs laser on the thermal-responsive shape-memory polymer to fabricate hierarchical micropillars, the laser-induced micropillars exhibited superhydrophobicity performance and the switchable wetting state was achieved [232]. Professor Tour’s group changed the different controlled atmospheres of laser-induced graphene process to regulate the wetting state of the LIG surfaces [233]. When varying the atmosphere from oxidizing to reducing condition, a great switch in the water contact angle of the LIG surfaces from 0° (superhydrophilic, O_2_ or air) to > 150° (superhydrophobic, Ar or H_2_) was observed. The atmosphere-mediated superhydrophobicity of micro/nanostructures by laser processing Cu and the subsequent adsorption of hydrophobic volatile organics was also reported [152]. In conclusion, the chemical composition and surface morphology of micropatterns contributed to different wetting properties, which had significance for guiding application researches in electrochemical catalysis, energy storage and electronic devices.

#### Laser Streaming

Transforming a laser beam into a mass flow derived additional applications in laser propulsion, microfluidics and laser surgery and cleaning [234]. Wang et al. firstly proposed the optofluidic concept, demonstrating the successful generation of high-speed liquid flow by pulsed laser streaming on a glass window after surface decorating with high-density Au nanoparticles [235]. The optofluidic mechanism was demonstrated that plasmonic nanoparticles generated ultrasonic waves via the photoacoustic effect when a pulsed laser was incident, and then, the ultrasonic waves pushed their surrounding media to generate laser streaming. Then after using the gold-implanted plasmonic quartz plate to replace the glass cuvette with Au nanoparticles, similar photoacoustic laser streaming occurred and the mechanism of the photoacoustic effect and vibration propagation was verified [236]. This laser-driven optofluidic strategy will provide an opportunity for versatile microfluidic applications.

Therefore, the laser microfabrication process, especially the “cold microfabrication” process, allows precise control of the processing sites. Therefore, laser microfabrication realizes mask-free patterning, which benefits for the construction of hierarchical structures on flexible substrates. It is noted that although the laser microfabrication process is significantly fast and time saving when line-by-line scanning, the laser punch treatment or point-by-point exposure treatment is time consuming, because that laser process is not a parallel processing technique compared with UV lithography. Alternatively, the use of a patterned laser source or multi-laser beams can substantially enhance the processing efficiency. In summary, the laser technology is expected to contribute to the advancing fabrication of different electronic devices, such as supercapacitors, batteries, sensors, electrocatalytic electrodes and other application systems.

## Outlook and Future Challenges

Laser techniques operating with pulsed or continuous-wave modes covering the wavelength ranges of ultraviolet, visible, and infrared have been employed in synthesis or microfabrication of materials. In terms of the synthesis of nanomaterials by laser, the basic processing principle is that the absorption of laser irradiation causes the photothermal or/and photochemical effects, which drive the conversion and crystallization of the precursor materials. The demanded thermal or chemical effect can be tuned by varying the laser process parameters, such as the scanning rate, pulse width and laser intensity. Thus, the choice of laser wavelength is related to the light absorption properties of the precursor materials. If photothermal effects predominate during the synthesis process, laser with a long wavelength is essential, such as the CO_2_ laser, especially for the laser synthesis of carbon materials. In terms of microfabrication of nanostructures by laser technology, two kinds of microfabrication styles are included, one is primarily based on the laser-induced photothermal effect through synthesis of the purpose materials at a localized position to construct the patterned nanostructures. The other is primarily based on the sputtering etching effect to obtain the desired nanostructure pattern. The feature of localized synthesis to construct the patterned nanostructures is similar to that of laser synthesis, and the only difference is that the formation of the pattern is strongly dependent on the laser process parameters, especially the pulse width, while the sputtering etching effect to obtain the desired nanostructured pattern is a so-called cold microfabrication process, which needs highly focused energy to avoid the burrs induced by photothermal effects. Therefore, the ultrafast laser is in ascendancy. Because that the ultrafast laser possesses a high intensity and ultrashort pulse width, thermal side effects are minimized and nonlinear absorption can take place in almost any material. As a result, brittle and transparent materials such as glasses can be processed, and because there is no heat exchange during the ultrafast pulsed laser processing, collateral damage and thermal stress are minimized. Thus, a more precise patterned nanostructure can be obtained.

As an alternative to the conventional annealing treatment, laser processing offers promising advantages. Firstly, this process is significantly fast and time saving. As experimentally verified, laser processing brings the heating and cooling rates of > 106 °C s^−1^, which is orders of magnitude higher than that of the conventional thermal treatment. Therefore, rapid fabrication of materials with minimal energy losses becomes possible. Secondly, the laser microfabrication process, especially the “cold microfabrication” process, allows precise control of the processing sites. Therefore, laser microfabrication realizes mask-free patterning, which benefits for the construction of hierarchical structures on flexible substrates. Thirdly, laser processing is adaptable for large size and roll-to-roll manufacturing and compatible with a variety of materials. The roll-to-roll manufacturing can be realized by integrating and digitizing the laser process parameters with computer design and a manufacturing system. Lastly, due to the advantage of precise control of the laser process, the in situ synthesis or in situ sputtering etching, which retaining the precursor morphology after laser processing, can be tailored by tuning the laser focus distance. Hence, laser synthesis and microfabrication are promising protocols for developing nanomaterials and nanostructures for various applications.

In spite of the advantages discussed above, laser processing of nanomaterials still confronted with several limitations. In terms of laser microfabrication for patterning, although this process is significantly fast and time saving when line-by-line scanning, the laser punch treatment or point-by-point exposure treatment is time consuming, because that laser process is not a parallel processing technique compared with UV lithography. In addition, 3D nanostructures are difficult to construct through laser processing. Alternatively, the use of a patterned laser source or multi-laser beams can substantially solve both of the above-mentioned issues. For practical production, the processing efficiency can be enhanced by adapting holographic laser processing or spatial light modulators. In the future, with the rapid development of parallel laser processing techniques or by combining 3D printing program, laser fabrication of 3D nanostructures can be completed in a more efficient manner. In addition, although various nanostructure patterns can be fabricated through the laser process, more nanostructure styles have not been realized compared with those constructed by wet chemical methods. Therefore, in situ transformation from the preconstructed nanostructures through the thermal effect of the unfocused laser is also a potential proposal for expanding the application of laser process.

Laser as a synthetic technique and/or microfabrication technique is not limited to the aforementioned applications. Laser synthesis and microfabrication have great potential for many other development directions. For instance, more compound species can be controllably synthesized by laser ablation under different atmospheres instead of only air or Ar atmosphere, such as the synthesis of nitrides under the N_2_ or NH_3_ atmosphere, synthesis of sulfides under the H_2_S atmosphere, synthesis of carbides under the CH_4_ atmosphere and even synthesis under the CO_2_ atmosphere for oxides or carbides with unique properties. In addition, the limited resolution of the laser process in nanomaterial processing should be improved. By introducing different optical schemes together with the integration with other systems, controlled laser fabrication with higher precision can be achieved. Besides, the combination of laser cold processing and laser thermal synthesis is conducive to the realization of more sophisticated electronics with precise patterns and fine functionalization. In consequence, with the rapid development of laser processing technology, additional nanomaterial-based applications will be explored. With the continuous efforts that are being devoted to this dynamic field, laser as a synthetic technique and/or microfabrication technique will have broad application prospects.

## Abbreviations

GO: Graphene oxide
rGO: Reduced graphene oxide
G: Graphene
GF: Graphene foam
DVD: Digital video disk
TE: Transverse electric
DP: Dielectric permittivity
TE-SPs: Transverse electric mode surface plasmons
Nd-YAG: Neodymium-doped yttrium aluminum garnet
PI: Polyimide
LIG: Laser-induced graphene
LIGP: Laser-induced graphene paper
LSG: Laser-scribed graphene
LOM: Laminated object manufacturing
3D: Three dimensional
PTFE: Polytetrafluoroethylene
CNS: Carbon nanospheres
CVD: Chemical vapor deposition
EG: Epitaxial growth
SiC: Silicon carbide
DLC: Diamond-like carbon
PLD: Pulsed laser deposition
OES: Optical emission spectroscopy
LIF: Laser-induced fluorescence
PET: Polyethylene terephthalate
PBI: Polybenzimidazole
DMSO: Dimethyl sulfoxide
ITO: Indium tin oxide
FTO: F-doped tin oxide
LIBs: Lithium-ion batteries
MOFs: Metal–organic frameworks
ZIF-67: Zeolitic imidazolate frameworks
MNPs: Metal nanoparticles
nano-LaMP: Nanoscale laser metallurgy and patterning
TMCs: Transition metal carbide nanoparticles
MCG: Molybdenum carbide–graphene
TMDs: Transition metal dichalcogenides
LIAS: Laser irradiation-assisted selenization
PSCs: Perovskite solar cells
LAL: Laser ablation in liquids
LFL: Laser fragmentation in liquids
LML: Laser melting in liquids
PLAL: Pulsed laser ablation of a solid target in liquids
PLICN: Pulsed laser irradiation of colloidal nanoparticles in liquids
AuNPs: Gold nanoparticles
fs: Femtosecond
LDHs: Layered double hydroxides
TONs: Ternary oxide nanocrystals
SAAs: Single-atom alloys
Cl-CDs: Cl-functionalized carbon dots
CB: Conduction band
N-CNTs: N-doped carbon nanotubes
MWCNT: Multi-walled carbon nanotube
UV: Ultraviolet
Vis: Visible
NIR: Near infrared
CW: Continuous wave
SSG: Solar steam generation
OPN: Optothermally gated photon nudging
HOPGF: Highly vertically ordered pillar array of graphene framework
EES: Electrochemical energy storage
SCs: Supercapacitors
MSCs: Micro-supercapacitors
PANI: Polyaniline
LaCVP: Laser-assisted chemical vapor pyrolysis
LDW: Laser direct writing
RH: Relative humidity
TENG: Triboelectric nanogenerator
OLED: Organic light-emitting device
BJTs: Bipolar junction transistors
OTNL: Optothermoplasmonic nanolithography
HER: Hydrogen evolution reaction
OER: Oxygen evolution reaction
ORR: Oxygen reduction reaction
LIG-O: Oxidized laser-induced graphene
LIG-A: Annealed laser-induced graphene
GC electrode: Glassy carbon electrode
